# The Human Brain Is Best Described as Being on a Female/Male Continuum: Evidence from a Neuroimaging Connectivity Study

**DOI:** 10.1093/cercor/bhaa408

**Published:** 2021-01-20

**Authors:** Yi Zhang, Qiang Luo, Chu-Chung Huang, Chun-Yi Zac Lo, Christelle Langley, Sylvane Desrivières, Erin Burke Quinlan, Tobias Banaschewski, Sabina Millenet, Arun L W Bokde, Herta Flor, Hugh Garavan, Penny Gowland, Andreas Heinz, Bernd Ittermann, Jean-Luc Martinot, Eric Artiges, Marie-Laure Paillère-Martinot, Frauke Nees, Dimitri Papadopoulos Orfanos, Luise Poustka, Juliane H Fröhner, Michael N Smolka, Henrik Walter, Robert Whelan, Shih-Jen Tsai, Ching-Po Lin, Ed Bullmore, Gunter Schumann, Barbara J Sahakian, Jianfeng Feng, for the IMAGEN consortium

**Affiliations:** 1 Shanghai Centre for Mathematical Sciences, Fudan University, Shanghai, 200433, China; 2 Institute of Science and Technology for Brain-Inspired Intelligence, Ministry of Education-Key Laboratory of Computational Neuroscience and Brain-Inspired Intelligence and Research and Research Institute of Intelligent Complex Systems, Fudan University, Shanghai, 200433, China; 3 State Key Laboratory of Medical Neurobiology and Ministry of Education Frontiers Center for Brain Science, Institutes of Brain Science and National Clinical Research Center for Aging and Medicine, Huashan Hospital, Fudan University, Shanghai, 200433, China; 4 Institute of Cognitive Neuroscience, School of Psychology and Cognitive Science, East China Normal University, Shanghai 200062, China; 5 Department of Psychiatry, University of Cambridge, Cambridge, CB2 0SZ, UK; 6 Behavioural and Clinical Neuroscience Institute, University of Cambridge, Cambridge, CB2 3EB, UK; 7 Medical Research Council-Social, Genetic, and Developmental Psychiatry Centre, Institute of Psychiatry, Psychology, and Neuroscience, King's College London, London, SE5 8AF, UK; 8 Department of Child and Adolescent Psychiatry and Psychotherapy, Central Institute of Mental Health, Medical Faculty Mannheim, Heidelberg University, Mannheim, 69117, Germany; 9 Discipline of Psychiatry, School of Medicine and Trinity College Institute of Neuroscience, Trinity College Dublin, Dublin, D02 PN40, Ireland; 10 Department of Cognitive and Clinical Neuroscience, Central Institute of Mental Health, Medical Faculty Mannheim, Heidelberg University, Manheim, 69117, Germany; 11 Department of Psychology, School of Social Sciences, University of Mannheim, Mannheim, 68131, Germany; 12 Departments of Psychiatry and Psychology, University of Vermont, Burlington, VT 05405, USA; 13 Sir Peter Mansfield Imaging Centre School of Physics and Astronomy, University of Nottingham, University Park, Nottingham, NG7 2RD, UK; 14 Department of Psychiatry and Psychotherapy, Campus Charité Mitte, Charité, Universitätsmedizin Berlin, Berlin, 10117, Germany; 15 Physikalisch-Technische Bundesanstalt (PTB), Abbestraße 2, 10587 Berlin, Germany; 16 Institut National de la Santé et de la Recherche Médicale, INSERM U1299 ``Developmental trajectories & psychiatry''; Université Paris-Saclay, Ecole Normale supérieure Paris-Saclay, CNRS, Centre Borelli; 91190 Gif-sur-Yvette, France; 17 Etablissement Public de Santé (EPS) Barthélemy Durand, 91700 Sainte-Geneviève-des-Bois, France; 18 Assistance Publique—Hêpitaux de Paris, Department of Child and Adolescent Psychiatry, Pitié-Salpêtrière Hospital, Paris, 75006, France; 19 Institute of Medical Psychology and Medical Sociology, University Medical Center Schleswig Holstein, Kiel University, Kiel, 24118, Germany; 20 Department of Child and Adolescent Psychiatry and Psychotherapy, University Medical Center Göttingen, Göttingen, 37075, Germany; 21 Clinic for Child and Adolescent Psychiatry, Medical University of Vienna, Vienna, 1090 Wien, Austria; 22 Department of Psychiatry and Neuroimaging Center, Technische Universität Dresden, Dresden, 01087, Germany; 23 School of Psychology and Global Brain Health Institute, Trinity College Dublin, Dublin, D02 PN40, Ireland; 24 Department of Psychiatry, Taipei Veterans General Hospital, Taipei, 11217, Taiwan; 25 School of Medicine, National Yang-Ming University, Taipei, 11221, Taiwan; 26 Institute of Neuroscience, National Yang-Ming University, Taipei, 11221, Taiwan; 27 Department of Computer Science, University of Warwick, Coventry, CV4 7AL, UK; 28 Collaborative Innovation Center for Brain Science, Fudan University, Shanghai, 200433, China; 29 Cambridgeshire and Peterborough National Health Service (NHS) Foundation Trust, Huntingdon, CB21 5EF, UK; 30 PONS Research Group, Department of Psychiatry and Psychotherapy, Campus Charité Mitte, Charitéplatz 1, Berlin, 10117, Germany; 31 PONS Centre, Institute of Science and Technology for Brain-Inspired Intelligence, Fudan University, Shanghai, 200433, China

**Keywords:** androgyny, brain functional network, sex difference

## Abstract

Psychological androgyny has long been associated with greater cognitive flexibility, adaptive behavior, and better mental health, but whether a similar concept can be defined using neural features remains unknown. Using the neuroimaging data from 9620 participants, we found that global functional connectivity was stronger in the male brain before middle age but became weaker after that, when compared with the female brain, after systematic testing of potentially confounding effects. We defined a brain gender continuum by estimating the likelihood of an observed functional connectivity matrix to represent a male brain. We found that participants mapped at the center of this continuum had fewer internalizing symptoms compared with those at the 2 extreme ends. These findings suggest a novel hypothesis proposing that there exists a neuroimaging concept of androgyny using the brain gender continuum, which may be associated with better mental health in a similar way to psychological androgyny.

## Introduction

In an ever-changing global environment, new learning for successful adaptation requires that we are able to be attentive to the world around us, cognitively flexible and able to employ a wide range of strategies. The ability to rapidly understand external context and decide on the optimal response, within a specific context, better enables us to take advantage of time-limited opportunities and thus provides us with mastery over the situation, thereby instilling resilience. Therefore, adaptiveness of cognition and behavior confers an advantage for individuals. Those who are limited by restricted approaches, stereotyped responses, and excessive internalizing in a variety of situations, including social, educational, and occupational ones, are less likely to flourish in society. It has been shown that being at the extreme end of the male continuum is disadvantageous both socially and psychologically. For example, these detrimental effects have been well-evidenced by a meta-analysis of 78 studies of about 20 000 participants, showing that conformity to typical masculine norms, for example, self-reliance and exercise of power over women, incurred social costs and psychiatric symptoms, including depression, loneliness, and substance abuse (Wong, et al. 2017). In contrast to these extreme stereotyped norms for males and females, “psychological androgyny” (Bem 1974, 1981, 1994) is the term that represents a flexibility and adaptability in sex roles and the behaviors associated with sex roles. An androgynous person possesses both masculine and feminine traits and the circumstances determine which traits (masculine or feminine) are employed (Rice 2006). Therefore, an androgynous person’s behavior is not influenced by a gender schema. Many psychological studies have suggested that psychological androgyny, which allows for more flexible behavioral responses may be beneficial to mental health (Vafaei et al. 2014, Juster et al. 2016, Pauletti et al. 2017). For example, psychological androgyny was associated with fewer internalizing problems (Pauletti et al. 2017), higher creativity (Norlander et al. 2000), and has been found to be psychoprotective (Prakash et al. 2010). There are many reports of differences between male and female brains in the literature (Ruigrok et al. 2014, Satterthwaite et al. 2014, Choleris et al. 2018, Jiang et al. 2020). However, whether an “androgynous” brain, with a well-balanced combination of both female and male features, offers better mental health compared to a brain with predominantly female or male features, remains an unanswered question.

Most of the previous studies have focused on identifying sex differences in the brain (Choleris et al. 2018), but the identified effect sizes were generally small and lacked significant behavioral association (Hines 2020). At the structural level, females had higher gray matter volume (GMV) in the middle frontal gyrus (Z_2186_ = 5.34) and lower GMV in the orbital frontal cortex (Z_2186_ = 5.07) (Ruigrok et al. 2014). At the functional level, females had a lower mean network positive-participation coefficient (Z_672_ = 2.21) (Satterthwaite et al. 2014). Although the effect size of those sex differences was small, multivariate classifiers have been trained to classify the sex of the brain (Satterthwaite et al. 2014, Weis et al. 2019) and achieved the best accuracy of 75% using independent test samples (for sample sizes ranging from 600 to 1700). These findings suggested that the brain’s functional architecture may have both female and male characteristics at the same time (Joel et al. 2015). Therefore, we hypothesized that the brain’s functional architecture can be mapped onto a continuum, and we used the biological information in regard to sex (male/female) to define the ends of the continuum. The importance of brain androgyny, akin to psychological androgyny, is that you are neither male nor female, but a combination of both. This gender continuum as a neuroimaging-defined marker of psychological androgyny may enable us to investigate the nonlinear relationships between brain gender and behavior or the variation in behavior within a sex group, which could not be uncovered using the biologically binary sex categories.

**Figure 1:**
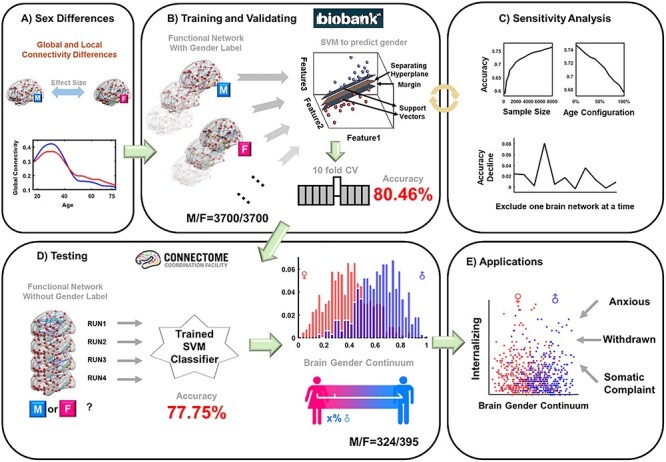
Study overview. (*A*) Both global and local brain functional connectivity were compared between 2 sex groups in different age groups; (*B*) based on the brain functional connectivity, a linear SVM to classify the female and the male brains was trained using the UKB sample with 10-fold cross-validation; (*C*) the effects of the sample size, the age composition and the functional networks on the SVM performance were assessed by sensitivity analyses; (*D*) the SVM model was tested using an independent sample from the HCP, and the test–retest reliability of the SVM prediction was also assessed among the 4 runs of the HCP data. A brain gender continuum was built by using the continuous output of the SVM classifier, with a value closer to 1 as more likely to be a male brain while a value closer to 0 as more likely to be a female brain; (*E*) the brain gender continuum was applied to the HCP participants and its associations with the internalizing and externalizing symptoms were assessed.

In this study, we used the resting-state functional magnetic resonance imaging (fMRI) data obtained from 9620 participants, who were aged between 17 and 78 years and recruited from 4 independent cohort studies. We investigated the sex differences of the brain functional networks in different age groups and systematically tested the potentially confounding effects on the identified differences. We subsequently built a multivariate classifier to estimate the likelihood of a given functional brain network to represent a male brain. Using this likelihood, we defined a brain gender continuum, and validated this definition by both sensitivity analyses and test–retest reliability analysis. Finally, as a demonstration of the behavioral relevance of the brain gender continuum, we tested the hypothesis that participants at the middle of this brain gender continuum, that is, with putatively androgynous brain network organization, had better mental health, specifically fewer internalizing or externalizing symptoms. Figure 1 shows the overall design of this study.

## Materials and Methods

### Participants

We used the following datasets to cover different age groups (Table 1): The UK Biobank (UKB) study (Miller et al. 2016)—a population-based cohort—recruited >500 000 participants in the United Kingdom, and 10 000 of them had neuroimaging data available. After the quality control (see Supplementary Method S1), we used the neuroimaging data from 7972 participants (age: 62.33 ± 7.49 years, M/F = 3733/4239, with mean framewise displacement [FD] <  0.3 mm). The Human Connectome Project (HCP; WU-Minn Consortium; the 900 Subject Release, S900) (Essen et al. 2013)—a neuroimaging cohort of healthy adults—had 877 participants with resting-state fMRI data, among which 823 had 4 scans. Our analyses focused on 719 participants (age: 28.81 ± 3.66 years, M/F = 324/395) had all 4 scans (823/877) and low head motion (mean FD < 0.3 mm, 719/823). The IMAGEN study—a population-based neuroimaging cohort (Schumann et al. 2010)—had 793 participants with quality controlled fMRI data (age: 19.33 ± 1.03 years, M/F = 381/412, mean FD < 0.3 mm). Notably, we also used the data from 136 healthy participants with a wider age range (age: 44.09 ± 12.03 years, M/F = 57/79, mean FD < 0.3 mm) recruited at the Department of Biomedical Imaging and Radiological Sciences and Brain Connectivity Laboratory, Institute of Neuroscience in National Yang-Ming University in Taipei, namely the Yang-Ming University (YMU) dataset (Yao et al. 2013).

**Table 1:** Demographics of all datasets used in our study

Datasets		Sample size	Age (years)	Internalizing symptom	Externalizing symptom
IMAGEN	Overall	793	19.33 ± 1.03	–	
	Female	412	19.33 ± 0.99	–	
	Male	381	19.33 ± 1.07	–	
HCP	Overall	719	28.81 ± 3.66	10.18 ± 8.57 (*n* = 691)	8.63 ± 6.30
	Female	395	29.42 ± 3.49	10.07 ± 8.27 (*n* = 381)	7.43 ± 5.48
	Male	324	28.07 ± 3.73	10.32 ± 8.93 (*n* = 310)	10.10 ± 6.90
UK Biobank	Overall	7972	62.33 ± 7.49	-	
	Female	4239	62.93 ± 7.60	–	
	Male	3733	61.79 ± 7.34	–	
Yang-Ming University	Overall	136	44.09 ± 12.03	–	
	Female	79	45.82 ± 12.22	–	
	Male	57	41.68 ± 11.42	–	

The Internalizing and externalizing symptom used in HCP dataset was recruited from Achenbach Adult Self Report.

Each cohort study was approved by its corresponding ethics committee. All adult participants provided written informed consent after information on the research procedures had been provided by each cohort study team. For the IMAGEN study, when the children were under 18 years old, the children gave assent and their parents or legal guardian provided written informed consent.

### Imaging Acquisition

The UKB participants were scanned on a 3-T Siemens Skyra scanner (Munich, Germany) with a spatial resolution of 2.4-mm isotropic voxels, a repetition time (TR) of 0.735 s, and a echo time (TE) of 39 ms. Scanning was conducted in ~6 min. The HCP participants were scanned on a 3-T Siemens “customed Connectome Skyra” scanner in 2 sessions with two 15-min runs each and the main scanning parameters were 2.0-mm isotropic voxels, 0.72 s TR, 33.1 ms TE. The IMAGEN neuroimaging data were collected at 7 centers on 3-T scanners (Siemens used in Munich, Germany; Philips used in Best, The Netherlands; General Electric used in Chalfont St Giles, United Kingdom; Bruker used in Ettlingen, Germany), with a slice thickness of 2.4 mm, planar resolution of 3.4 mm, with a TR of 2.2 s, and a TE of 30 ms for about 6 min. The YMU images were collected on a Siemens Trio 3T scanner at the YMU, with a slice thickness of 3.4 mm, planar resolution of 3.44 mm, with a TR of 2.5 s, and a TE of 27 ms for about 8 min.

### Preprocessing

We used the Functional Magnetic Resonance Imaging of the Brain (FMRIB) Software Library (FSL, v5.0.10; Jenkinson et al. 2012) to preprocess UKB resting-state fMRI data. The preprocessing procedure included slice-timing correction, motion correction, spatial smoothing with a 6-mm full-width at half-maximum Gaussian kernel, and wavelet despiking. The averaged white matter, cerebrospinal fluid signal, and 24 motion parameters were then regressed out from a voxel-level time series. The functional image was subsequently registered to a T1 structural image and normalized to 3-mm standard MNI space using linear and nonlinear registration with the default parameters. The same preprocessing pipeline was applied to both the IMAGEN data and the YMU data. Details could be found in Supplementary Method S11.

The resting-state functional images downloaded from the HCP consortium that already underwent HCP’s minimal preprocessing pipeline (Glasser et al. 2013). This pipeline mainly included the corrections for gradient-nonlinearity-induced distortion, head motion, and B_0_ distortion, and the transformation of the corrected fMRI data to a 2 mm Montreal Neurological Institute (MNI) space. All of the transforms for each registration and distortion correction step were concatenated and applied in a single resampling step. Next, the global intensity normalization and a brain mask were applied. Finally, the structured artifacts were removed by an ICA (independent component analysis) + FIX (FMRIB’s ICA-based X-noisifier) denoising procedure that was specifically trained on HCP data (Smith et al. 2013). The head motion parameters were also regressed out of the data (Satterthwaite et al. 2013). The downloaded data were ready for conventional volume-based analyses (Glasser et al. 2013). These preprocessing steps were carried out by the HCP consortium using FSL (FMRIB Software Library), FreeSurfer and the Connectome Workbench software. More details are provided in the WU-Minn HCP 1200 Subjects Data Release Reference Manual (https://www.humanconnectome.org/storage/app/media/documentation/s1200/HCP_S1200_Release_Reference_Manual.pdf).

The region-of-interest (ROI)-level time series based on the Anatomical Automatic Labelling parcellation (version 2) (AAL2 parcellation) (Rolls et al. 2015) were finally extracted by averaging the voxel-level time series within each of the AAL2 regions. ROI-level time series based on Power’s parcellation (Power et al. 2011) were also extracted for validation tests.

### Behavioral Assessment

The age of menopause (Field ID:3581) reported by the UKB participants (*n* = 2565) during their imaging visit were used in the current analysis. The Achenbach Adult Self-Report (ASR) (Achenbach 2009) was assessed for the participants in HCP. This assessment has been widely used for adults aged 18–59 years, including 123 items for the behavioral, emotional, and social problems. Anxious, withdrawn, and somatic complaints comprise the internalizing dimension, whereas aggressive, rule-breaking, and intrusive behaviors comprise the externalizing dimension. Finally, 691 subjects (M/F = 310/381) with complete family information and ASR scores were included in the current study.

### Statistical Analyses

#### Group Comparison

We first studied the sex difference of functional connectivity at multiple levels in different age groups. First, a functional connectivity between each pair of brain regions was calculated. Second, at the network level, the mean connectivity of the functional connectivity among the brain regions within a brain functional network was used. In Power’s parcellation (Power et al. 2011), whole brain was divided into 11 functional networks, including sensory/somatomotor network, cingulo-opercular task control network, auditory network, default mode network, memory retrieval network, visual network, frontal–parietal task control network, salience network, subcortical, ventral attention network, and dorsal attention network. Third, we examined the global connectivity by averaging all functional connectivity.

We used student’s *t*-test to calculate the effect size (i.e., Cohen’s *d*) of the sex difference in cases with and without considering covariates, including total intracranial volume (TIV), signal to noise ratio (SNR), and mean FD. The seven data collection sites were considered as additional covariates in the IMAGEN analyses. Bootstrap with 100 000 repetitions was applied to these comparisons. A correlation analysis was applied to the statistics before and after controlling additional variables to study the influence of covariates. At the network level, the correlation analysis had 64 degrees of freedom (from 66 types of pairwise connections, among 11 brain networks, of which 55 were inter-network connections and 11 were intra-network connections). At the regional level, the correlation analysis had 4369 degrees of freedom (from 4371 functional connectivity among 94 brain regions). A localized spline curve was used to fit the trajectory of global functional connectivity (gFC) with age. The fitted values for spline curve with and without considering the covariates were calculated for each age in the age range of 17–78 years old, with a manually chosen step length of 0.2 years. A correlation analysis with a degree of freedom of 304 was applied to those 2 groups of fitted values to assess the effect of covariates to the trajectory of gFC.

#### Multivariate Classifier

We used a support vector machine (SVM; Cortes and Vapnik 1995) with a linear kernel to classify the resting-state functional connectivity network into 2 sexes. We used the default box constraint parameters and the SMO (sequential minimal optimization) solver (RongEn et al. 2005) to train the SVM classifier. All of the functional connectivity data were used as the input features without feature selection. We randomly chose 3700 male subjects and 3700 female subjects from the UKB dataset to train our model; their age and corresponding squared and cubed age was regressed out from their functional connectivity. All functional connectivity was used as classifier features without feature selection. To assess the reliability of our model, we implemented a 10-fold cross-validation strategy on the UKB dataset. Next, all 7400 subjects were used to train a new SVM classifier and the classifier was tested using an independent data set, namely the HCP sample. The SVM classifier can provide the posterior probability computed through a sigmoid function. Using the coefficients derived from model fitting, the maximum likelihood estimate of the posterior probability $=\frac{1}{1+\exp \Big( AX+B\Big)}$, where *A* and *B* are coefficients, and *X* is the training data (Platt 1999). The posterior probability represents the probability that an input resulted in a certain output label, and we call that the “output scores of the SVM.” To understand the meaning of the weights of the functional connectivity in the trained SVM, we first tested the correlation between the SVM weights and the effect sizes of the sex-differences estimated by the group comparisons above. Second, we compared the FC at the network level among 3 groups of participants with the lower (<0.35), intermediate (0.35 ≤ gender ≤ 0.65) and higher (>0.65) output scores by the SVM (i.e., a number between 0 and 1) to see whether the output scores could be used to indicate the brain functional connectivity. Here, we compared all 66 types of pairwise connections among 11 brain networks.

#### Sensitivity Analysis

We tested the effects of the sample size, percentage of elderly subjects, and brain network features on the performance of the classifier built using the UKB sample. In the sensitivity analysis for sample size, we randomly selected subjects with different sample size from the UKB sample, ranging from 200 to 7000, and then compared the classification accuracies on the independent test data set (i.e., the HCP sample). In the sensitivity analysis for age, we changed the percentage of elderly subjects (i.e., older than 65 years old) in the training data set, whereas the training sample size was always fixed at 3000. For example, if the percentage of elderly subjects was 20%, then we randomly selected 2400 subjects from the younger group and 600 subjects from the older group. The 11 brain networks were defined by the Power’s parcellation (Power et al. 2011). In the sensitivity analysis for brain networks, we excluded the functional connectivity of one brain network from the SVM classifier at a time (i.e., excluding all the regions in a given network and all the functional connectivity attached to one of these regions), and then we compared the classification accuracies before and after excluding a network. Since the training samples were randomly chosen from the UKB, each test was repeated 100 times. The model performance was tested using the HCP dataset. The top 5% weights with the highest absolute values of the functional connectivity in the SVM classifier trained from UKB subjects were summarized into each functional brain network, that is, the networks with higher weights had greater difference between the 2 sex groups. To test whether these findings were atlas-dependent, we compared the results given by the AAL2 parcellation and the Power’s parcellation.

#### Behavior Association

Using the continuous SVM output between 0 and 1, we mapped each brain functional architecture onto a “gender continuum” with the value 1 as the most likely to be collected from a male brain and the value 0 as the most likely collected from a female brain. We tested the associations of the gender continuum with internalizing symptoms, considering biological sex, age, handedness, and head motion as covariates. The partial Pearson correlation was used and when assessing the quadratic relationship, the linear term was also used as an additional covariate. The results were plotted using BrainNet Viewer (Xia et al. 2013).

#### Significance Test

Considering the family relatedness in the HCP dataset, we applied the multilevel block-permutation analysis for linear models (PALM) (Winkler et al. 2014) to assess the significance (Winkler et al. 2015). The significance level, *p.perm*, was given by 100 000 random permutations. The false discovery rate (the Benjamini–Hochberg method) was used to correct for multiple comparisons among 2 sex groups, the linear and quadratic terms, and the internalizing and externalizing terms, denoted as *p.fdr*.

#### Code Availability

Code used in the study could be found at https://github.com/zy-fdu/Brain-Gender-Continuum.

## Results

### Sex Differences in the Brain Functional Architecture Were Age-Dependent

**Figure 2:**
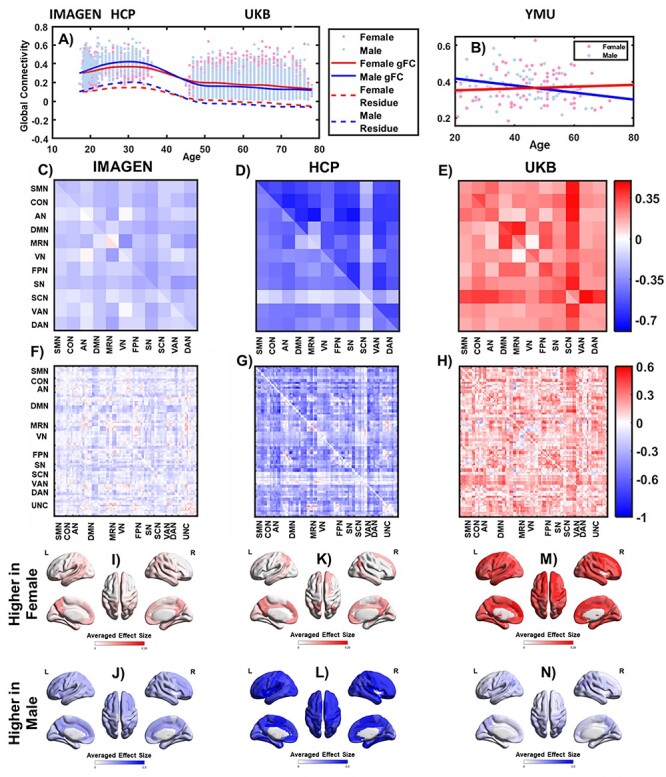
Brain sex differences. (*A*) Trajectories of the gFC in the female (red) and the male (blue) brains. Each dot stands for one individual. The lines were fitted by splines. After regressing the covariates, including the mean FD, SNR, and TIV, the trajectories of the residuals were reported as the dotted lines. In total 3 data sets (i.e., IMAGEN, HCP, and UKB) covered 3 age groups. (*B*) Trajectories of the gFC using the data collected at a single site covering a wider age range (i.e., the Yang-Ming data set). (*C*–*E*) Sex differences in 3 datasets at the network level. The mean of intra/inter-network connectivity was reported; (*F*–*H*) Sex differences in 3 datasets at the edge level. The brain regions were arranged into the brain functional networks. The sex-difference was reported for the connectivity between each pair of brain regions. In (*C*–*H*), the upper right triangle shows the original effect size, and the lower left triangle shows the effect size after controlling for the potential confounding factors (e.g., mean FD, SNR, and the TIV); (*I*–*N*) sex differences at the regional level. A negative sex difference meant this connectivity was stronger in the female brains compared with the male brains. The negative differences were averaged in (*I*), (*K*), (*M*) and the positive differences were averaged in (*J*), (*L*), (*N*).

In the global mean of the brain functional connectivity, during both adolescence (the IMAGEN study; Schumann et al. 2010) and young adulthood (the HCP; Essen et al. 2013), males had stronger gFC compared with females (Cohen’s *d*: 0.22, 95% CI: 0.08–0.36 for IMAGEN participants, and *d* = 0.56; 95% CI: 0.41–0.72 for HCP participants.), whereas elderly females showed higher gFC compared with elderly males ($d=-0.32$; 95% CI: −0.37 to −0.28; the UKB [Miller et al. 2016]; Fig. 2*A*). The trajectories of the gFC were validated using the YMU dataset (Yao et al. 2013; Fig. 2*B*; Cohen’s *d*: 0.5525, 95% CI: 0.08–1.08 for participants younger than 45 years of age, and *d* = −0.34; 95% CI: −0.83 to 0.14 for participants older than 45 years of age).

At the network level, we found that males had stronger functional connectivity compared with the females with small-to-medium effect sizes within the default mode network (DMN) ($d=0.21$), salience attention network (SN; $d=0.30$), and fronto-parietal task network (FPN; $d=0.24$), in the adolescent sample from IMAGEN (Fig. 2*C*). The effect sizes of these differences increased from small-to-medium to large in the adult sample from HCP as $d=0.58$ for DMN, 0.66 for SN, and 0.55 for FPN (Fig. 2*D*). After middle age, the functional connectivity decreased more steeply in males compared with females, so that the signs of sex differences were flipped, for example, $d=-0.38$ for DMN, −0.30 for SN, and −0.35 for FPN, in an older sample from UKB (Fig. 2*E*). The majority of the sex differences identified followed the same pattern that they were stronger in males compared with females in adolescents and adults, but after the middle age, they became stronger in females compared with males (see Supplementary Table S1).

In the single-center, lifespan sample from YMU, we divided the sample into the younger group (F/M = 37/30, age < 45 years old) and the older group (F/M = 42/27, age $\ge$ 45 years old). We found that the effect sizes of the sex-differences in the brain functional network connectivity (see Supplementary Table S2) were significantly correlated between the younger group and both the IMAGEN ($r=0.43,p=7.19\times{10}^{-7}$) and the HCP samples ($r=0.36,p=4.14\times{10}^{-5}$), and also between the older group and the UKB sample ($r=0.36,p=4.28\times{10}^{-5}$). The majority of sex-differences in the brain functional connectivity followed the same lifespan pattern, whereby the functional connectivity was stronger in males in the younger group (e.g., DMN: *d* = 0.65; SN: 0.49; FPN: 0.60) and became stronger in females in the older group (e.g., DMN: *d* = −0.39; SN: −0.52; FPN: −0.56).

At the regional level, averaging all the pair-wise correlations or edge weights for each brain regional node, we found that for adolescents (Fig. 2*I* and *J*) and adults (Fig. 2*K* and *L*) the weighted degree of the cingulate cortex had the greatest sex-difference (see Supplementary Table S3). Although for older subjects (Fig. 2*M* and *N*) the weighted degree of the angular gyrus and the precuneus had the greatest sex-differences (see Supplementary Table S4).

### No Significant Confounding Effect Was Identified

At global level, the trajectory fitted for gFC was almost identical to that fitted for the residual gFC after regressing out additional confounders, including the mean framewise-displacement (mean FD), SNR and TIV ($r=1.00, df=304$, Fig. 2*A*). At both the global network level (IMAGEN: $r=1.00$, Fig. 2*C*; HCP: $r=0.98$, Fig. 2*D*; UKB: $r=0.97$, Fig. 2*E*; *df* = 64) and the nodal level (IMAGEN: $r=0.99$, Fig. 2*F*; HCP: $r=0.89$, Fig. 2*G*; UKB:$r=0.67$, Fig. 2*H*; *df* = 4369), the effect sizes of the sex differences were correlated between those analyses with or without controlling for the covariates.

### Brain Gender Continuum Built by a Multivariate Model

**Figure 3:**
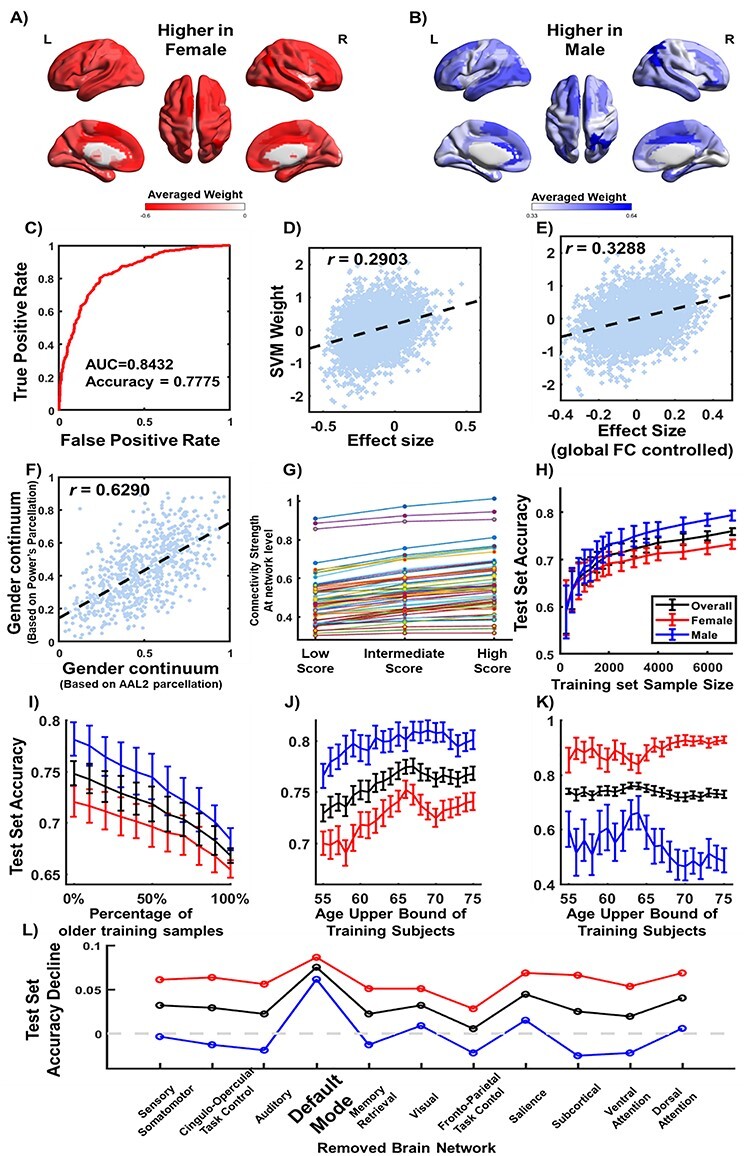
A multivariate classifier for sex based on the resting-state functional connectivity. (*A*) The negative weights in the SVM were averaged for each brain region among all its functional connectivity with the other brain regions. A negative weight meant this functional connectivity was in average stronger in the female brains compared with the male brains; (*B*) the positive weights in the SVM were averaged for each brain region; (*C*) performance of the SVM measured by the receive-operating characteristic (ROC) curve. Scatter plot of the SVM weights against the effect sizes of sex difference using the UKB sample before (*D*) and after (*E*) regressing out the gFC; (*F*) scatter plot of gender continuum calculated from Power Atlas (264 regions) and AAL2 Atlas (94 regions); (*G*) comparison of each of the intra- and inter-network connectivity (in total 66 types of connections) among the participants with the lower, higher and intermediate brain gender continuum scores. Influences on the classification accuracy of the trained SVM in the test sample when (*H*) changing the sample size; (*I*) changing the age composition while fixing the sample size at 3000; (*J*) changing the upper age bound of the participants, of the training data; (*K*) performance of SVM model without regressing age and its higher order terms, the model fails in male test samples; (*L*) removing one functional network from the input feature of the SVM.

We trained a SVM classifier that reached a 10-fold cross-validated accuracy of 80.46% within the UKB sample and reached a test accuracy of 77.75% (AUC = 0.84; Fig. 3*C*) using the independent HCP sample (accuracy of 4 HCP runs ranged from 69.26% to 73.30%, and the test–retest reliability of the SVM scores was high as the correlation of the SVM scores among these 4 runs ranged between 0.56 and 0.62 [*df*= 717]; see Supplementary Fig. S2).

The feature weights in the trained SVM were consistent with the sex difference detected by group comparison in the previous section. In the training sample (i.e., UKB; *n* = 7400; F/M = 3700/3700), we found that the SVM weights (Fig. 3*A* and *B*) were correlated with the effect sizes estimated by the group comparisons above ($r=0.29, df=4369$; Fig. 3*D*). As the SVM was a multivariate approach, the SVM weight of each functional connectivity was established while controlling for the contributions of other functional connectivity. Indeed, we found the correlation between the SVM weight and the effect size of the sex difference became significantly stronger after controlling for the gFC in the group comparison for each functional connectivity ($r=0.33, df=4369$; 95% CI of the correlation increase: 0.03–0.05; Fig. 3*E*).

Two extreme ends of the brain continuum represent brains with either predominantly female features or predominantly male features, compared with the center of this continuum. We found that the participants who were scored intermediately by the SVM had the intermediate connectivity strength at the network level. For example, in the testing sample (i.e., the HCP cohort of young adults, *n* = 719), the DMN connectivity was the highest at the right end with predominantly male features (brain gender score > 0.65, F/M = 32/153), lowest at the left end with predominantly female features (brain gender score < 0.35, F/M = 161/14), and intermediate in the middle of this continuum (0.35 < brain gender score < 0.65, F/M = 202/157; Fig. 3*G*). One-way analysis of variance showed the difference in the DMN connectivity among these 3 groups was significant (${F}_{\mathrm{688,2}}=13.8;p. fdr=1.7\times{10}^{-6}$ after correcting for multiple comparisons among all within-network and between-networks connectivity). The post-hoc comparisons confirmed that such differences were significant between the middle group and both the left (${t}_{532}=-3.21,p. fdr=0.002$) and the right groups (${t}_{542}=2.82,p. fdr=0.006$).

Controlling for age, the association between the number of years after menopause and the brain gender continuum score in the UKB female participants was significant but small ($r=0.048; df=2563$; 95% CI: 0.0082–0.0880). This result might suggest that as the sex hormone levels decrease, the brain gender continuum score, in females, moves towards the male end.

### Sensitivity Analysis of the Multivariate Model

We trained and tested a new SVM by regressing out the additional covariates (e.g., mean FD, SNR, and TIV) from the training sample and the testing sample. We found that the output score of the new SVM was significantly correlated with the corresponding output scores given by the SVM without controlling for these additional covariates ($r=0.77; df=717$; see Supplementary Fig. S3*B*). When we controlled for the mean FD and SNR only, as these 2 variables were relevant to the quality of the images, the classification accuracy was 74.55% in the independent test sample (AUC = 0.83; see Supplementary Fig. S3*A*).

The output score of the SVM (i.e., the gender continuum) based on the AAL2 parcellation was significantly correlated with the SVM score based on the Power264 parcellation ($r=0.63;p=1.88\times{10}^{-80}; df=717$; Fig. 3*F*). Compared with the pure random distribution, we found that significantly more functional connectivity with the top 5% SVM weights were intra-network connections within DMN ($P=0.0139$), and this finding was consistent between the AAL2 parcellation and the Power264 parcellation (see Supplementary Fig. S1).

Furthermore, by increasing sample size of the training set (i.e., UKB), the test accuracy using the independent test sample (i.e., HCP) gradually reached a stable value around 75% after the sample size reached 2000 (Fig. 3*H*). By removing one brain functional network from the SVM, we found that removing DMN significantly decreased the test accuracy (95% CI: [−11.40%, −4.73%] by bootstrap; Fig. 3*L*). We also found that a greater percentage of the older participants (older than 65 years) in the training sample (sample size remained the same as 3000) was correlated with lower test accuracy (*r* = −0.99, *p* = 2.34×10^−9^, *df* = 9, Fig. 3*I*; more details were provided in Supplementary Method S4). If we did not regress out age and its higher order terms, the SVM failed to identify the male brains in the test sample (mean accuracy = 54.27% ± 5.99%) but was systematically biased to label more brains as female (mean accuracy = 89.32% ± 2.92%; Fig. 3*K*). Therefore, we regressed out the age effect in the following analysis.

### Brain Androgyny Associated with Fewer Internalizing Symptoms

**Figure 4:**
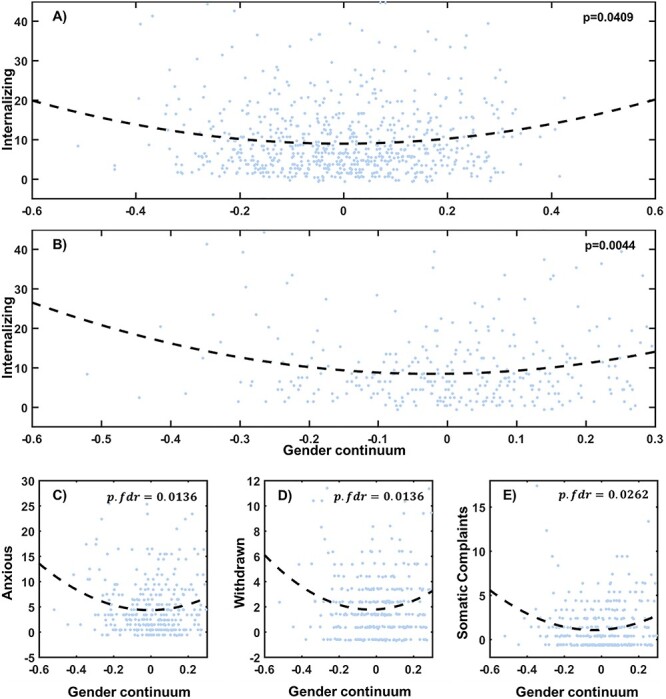
Behavioral association of the brain gender continuum. Scatter plot of the brain gender continuum score and the internalizing symptom score among (*A*) all participants; (*B*) male participants, from the HCP data set showed a U-shape relationship. The internalizing score reached its minima at a gender continuum score about 0.4922 and 0.6099, respectively. The scatter plots of the subscales of the internalizing symptom also showed a U-shape relationship, including anxious score (*C*), withdrawn score (*D*), somatic complaints (*E*). Residual of the brain gender continuum, as well as internalizing symptoms controlling mean FD, sex, age and headedness are plotted in the figure.

In the HCP cohort, we found that the internalizing score, but not the externalizing score, was associated with the second-order term of the brain gender continuum ($df=684;r=0.08;p. perm=0.0409,$uncorrected; Fig. 4*A*). This U-shaped relationship was mostly driven by the male participants (corrected among 2 sex groups, the internalizing and the externalizing symptoms, and the first and the second order terms; Fig. 4*B*). All 3 subscales of the internalizing symptoms followed the same relationship with the brain gender continuum in the male participants (anxious:$r=0.15,p. fdr=0.0136;$$\mathrm{withdraw}:r=0.16,p. fdr=0.0136;$$\mathrm{somatic}\ \mathrm{complaints}:r=0.13,p. fdr=0.0262$; Fig. 4*C*–*E*).

## Discussion

In the present study, we identified an age-dependent pattern of sex differences in the brain functional architecture using the fMRI data of nearly 10 000 participants from teenagers to older adults, and systematically examined the potentially confounding effects on these findings. Based on the identified sex differences, we trained an SVM classifier that achieved a 77.75% accuracy in an independent test sample. Using the continuous output of this SVM, we constructed a brain gender continuum and defined an androgynous brain to be at the middle of this continuum. Indeed, we showed that the patterns of functional connectivity, at the 2 extreme ends of this brain gender continuum, represented predominantly either more female or male features as compared with the center of the continuum. Finally, we used this brain gender continuum to uncover a U-shaped relationship between the neuroimaging-defined brain gender and mental health, particularly the participants with an androgynous brain indeed had fewer internalizing symptoms.

The age-dependency of the sex differences may be associated with a number of factors such as the behavior, genetics, and hormones. Research has shown that different environmental contexts, experiences, and behaviors, throughout the lifespan may alter the structural and functional architecture of the brain, in addition to modulation by neurotransmitters (Kolb and Gibb 2011). Genetic factors may also have differential expression across the lifespan, for example Deary et al. (2006) have shown different rates of heritability of intelligence across age. In addition, the sex hormones have nonlinear developmental trajectories (Haimov-Kochman and Berger 2014; Mcewen and Milner 2017) which increase during childhood and adolescence (Nottelmann et al. 1987) but decrease during aging (Rosario et al. 2004; Cui et al. 2013). Particularly, testosterone, a sex hormone, has been implicated in the developmental change of the DMN (Nota et al. 2016), and in our study we found that 3 brain regions (i.e., the cingulate cortex, angular cortex, and precuneus) with the most differences in their functional connectivity were all identified within the DMN and these differences were also supported by previous studies using smaller samples at different age groups (Lombardo et al. 2018; Ritchie et al. 2018; Ernst et al. 2019). Furthermore, in the trained SVM, a multivariate classifier, we also found that the DMN contributed the most to the classification accuracy of this model. Our findings suggest that the patterns of functional connectivity in the brain are unlikely to be entirely determined by the sex hormone levels. In the UKB sample, we showed that the greater the number of years since menopause, presumably reflecting decreased estrogen levels, the larger the gender brain continuum score, suggesting a shift towards the male end. However, the effect size of this association was small (*r* = 0.048). Therefore, while sex hormones influence the brain’s functional connectivity many other factors, including those discussed above, also have an impact.

After systematically testing the potential confounders, we confirmed the findings of sex differences in the brain’s functional connectivity. Based on the differences identified, we trained an SVM classifier and mapped each brain onto a brain gender continuum by using the continuous output of the SVM classifier. Some previous studies using cross-validation within the training samples achieved a high classification accuracy (~90%) (Wang et al. 2012; Luo et al. 2019). However, applying such classifiers to the independent test samples, only moderate classification accuracies could be achieved (~75%) (Satterthwaite et al. 2014; Weis et al. 2019), which were comparable with the classification accuracy of 77.75% achieved in the current study. Compared with the low classification accuracy (i.e., 65.7%) in a previous study using a test sample from a different age group compared with the training sample (Weis et al. 2019), our classifier achieved a better accuracy after regressing out age and its higher order terms from the functional connectivity matrix (77.75%). This result was in support of the finding that the sex difference in brain functional connectivity was age dependent.

The moderate classification accuracy of the multivariate classifier indicated that the brain functional architecture was unlikely to be conceptualized as binary, as is the case with biological sex, but was more likely to be continuously represented on a brain gender spectrum. At the behavioral level, Bem had hypothesized that an androgynous gender role would lead to higher self-esteem and better mental health (Bem 1974), since individuals identifying with androgyny are free to act in both masculine and feminine ways without many constraints of gender appropriateness (Bem 1977). In particular, the androgynous group reported having fewer internalizing symptoms (Pauletti et al. 2017). However, previous studies provided only the behavioral observations, therefore there was a need to understand the neural mechanism of such observations. Our results demonstrated that the participants whose brain functional connectivity mapped onto the androgynous segment of the brain gender continuum had fewer internalizing problems, which is advantageous for mental health. This U-shaped association was seen for both males and females, although it was most prominent in males. These findings may indicate that being more compassionate and sociable (traditionally female traits) could potentially improve self-esteem of men, thereby potentially reducing internalizing problems; but being more aggressive and confrontational (traditionally male traits) might not boost self-esteem of women (Pauletti et al. 2017). Future research should include self-report data on male/female behavioral traits within different contexts, for example work, home and social settings, which could further elucidate the relationship between psychological androgyny and the concept of brain androgyny.

However, the current study also has several limitations. First, no single large dataset exists that contains samples covering the entire lifespan, from infancy to old age. In our study, we first analyzed the large-scale multicenter samples from different age groups, and then validated the findings using a single-center sample covering a wider age range but with a smaller sample size. Across this age range, there will inevitably be many environmental factors which will have changed and may have some influence. Second, although the sex hormones have been implicated in the sex dimorphism of the brain’s functional architecture (Bao and Swaab 2011), we need the lifespan measurements of the sex hormones to further investigate the molecular mechanisms underlying the brain gender continuum.

## Supplementary Material

gender_continuum_supplement_bhaa408

## Notes

Dr Banaschewski has served as an adviser or consultant for Infectopharm, Lundbeck, Medice, Neurim Pharmaceuticals, Oberberg GmbH, and Shire; he has received conference support or speaking fees from Eli Lilly, Medice, and Shire; and he has received royalties from CIP Medien, Hogrefe, Kohlhammer, and Oxford University Press. Dr Bokde has received support from the Health Research Board (Ireland) and the National Children’s Foundation Tallaght, Tallaght University Hospital, Ireland. Dr Poustka has received speaking fees from Infectopharm and Shire; she receives funding from the Federal Ministry of Education and Research (BMBF), the German Research Foundation (DFG), and the European Union and royalties from Hogrefe, Kohlhammer, and Schattauer. Dr Sahakian has served as a consultant for Cambridge Cognition and Peak. During the preparation of this manuscript, Dr Luo was a visiting fellow at Clare Hall, Cambridge, United Kingdom. The other authors report no financial relationships with commercial interests.

## References

[ref1] Achenbach TM . 2009. Some needed changes in DSM - V: but what about children? Clin Psychol-Sci Pract. 16(1):50–53.

[ref2] Bao A , Swaab DF. 2011. Sexual differentiation of the human brain: relation to gender identity, sexual orientation and neuropsychiatric disorders. Front Neuroendocrin. 32(2):214–226. 10.1016/j.yfrne.2011.02.007 21334362

[ref3] Bem SL . 1974. The measurement of psychological androgyny. J Consult Clin Psychol. 42(2):155–162. 4823550

[ref4] Bem SL . 1977. On the utility of alternative procedures for assessing psychological androgyny. J Consult Clin Psychol. 45(2):196–205. 850004 10.1037//0022-006x.45.2.196

[ref38c] Bem SL . 1981. Gender schema theory: a cognitive account of sex typing. Psychol Rev. 88(4):354–364.

[ref38d] Bem SL . 1994. The Lenses of Gender: Transforming the Debate on Sexual Inequality. New Haven: Yale University Press.

[ref5] Choleris E , Galea LAM, Sohrabji F, Frick KM. 2018. Sex differences in the brain: implications for behavioral and biomedical research. Neurosci Biobehav Rev. 85:126–145. 29287628 10.1016/j.neubiorev.2017.07.005 PMC5751942

[ref6] Cortes C , Vapnik V. 1995. Support-vector networks. Mach Learn. 20(3):273–297.

[ref7] Cui J , Shen Y, Li R. 2013. Estrogen synthesis and signaling pathways during aging: from periphery to brain. Trends Mol Med. 19(3):197–209. 23348042 10.1016/j.molmed.2012.12.007 PMC3595330

[ref8] Deary IJ , Spinath FM, Bates TC. 2006. Genetics of intelligence. Eur J Hum Genet. 14(6):690–700. 16721405 10.1038/sj.ejhg.5201588

[ref9] Ernst M , Benson BE, Artiges E, Gorka AX, Lemaitre H, Lago T, Miranda R, Banaschewski T, Bokde ALW, Bromberg U, et al. 2019. Pubertal maturation and sex effects on the default-mode network connectivity implicated in mood dysregulation. Transl Psychiatry. 9(1):103. 30804326 10.1038/s41398-019-0433-6 PMC6389927

[ref10] Essen DCV , Smith SM, Deanna M, Behrens TEJ, Yacoub E, Ugurbil K. 2013. The WU-Minn human connectome project: an overview. NeuroImage. 80:62–79. 23684880 10.1016/j.neuroimage.2013.05.041 PMC3724347

[ref11] Glasser MF , Sotiropoulos SN, Wilson JA, Coalson TS, Fischl B, Andersson JLR, Xu J, Jbabdi S, Webster MA, Polimeni JR, et al. 2013. The minimal preprocessing pipelines for the human connectome project. NeuroImage. 80:105–124. 23668970 10.1016/j.neuroimage.2013.04.127 PMC3720813

[ref12] Haimov-Kochman R , Berger I. 2014. Cognitive functions of regularly cycling women may differ throughout the month, depending on sex hormone status: a possible explanation to conflicting results of studies of ADHD in females. Front Hum Neurosci. 8:191–191. 24744721 10.3389/fnhum.2014.00191 PMC3978296

[ref13] Hines M . 2020. Neuroscience and sex/gender: looking back and forward. J Neurosci. 40(1):37. 31488609 10.1523/JNEUROSCI.0750-19.2019 PMC6939487

[ref14] Jenkinson M , Beckmann CF, Behrens TE, Woolrich MW, Smith SM. 2012. FSL. NeuroImage. 62:782–790. 21979382 10.1016/j.neuroimage.2011.09.015

[ref15] Joel D , Berman Z, Tavor I, Wexler N, Gaber O, Stein Y, Shefi N, Pool J, Urchs S, Margulies DS, et al. 2015. Sex beyond the genitalia: the human brain mosaic. Proc Natl Acad Sci U S A. 112(50):15468–15473. 26621705 10.1073/pnas.1509654112 PMC4687544

[ref16] Juster R , Pruessner JC, Desrochers AB, Bourdon O, Durand N, Wan N, Tourjman V, Kouassi E, Lesage A, Lupien SJ. 2016. Sex and gender roles in relation to mental health and allostatic load. Psychosom Med. 78(7):788–804. 27359170 10.1097/PSY.0000000000000351

[ref17] Kolb B , Gibb R. 2011. Brain plasticity and behaviour in the developing brain. J Can Acad Child Adolesc Psychiatry. 20(4):265–276. 22114608 PMC3222570

[ref18] Lombardo MV , Auyeung B, Pramparo T, Quartier A, Courraud J, Holt R, Waldman J, Ruigrok ANV, Mooney N, Bethlehem RAI, et al. 2018. Sex-specific impact of prenatal androgens on social brain default mode subsystems. Mol Psychiatry. 25(9):2175–2188. 30104728 10.1038/s41380-018-0198-y PMC7473837

[ref19] Luo Z , Hou C, Wang L, Hu D. 2019. Gender identification of human cortical 3-D morphology using hierarchical sparsity. Front Hum Neurosci. 13:29. 10.3389/fnhum.2019.00029 PMC6374327 30792634

[ref20] Mcewen BS , Milner TA. 2017. Understanding the broad influence of sex hormones and sex differences in the brain. J Neurosci Res. 95(95):24–39. 27870427 10.1002/jnr.23809 PMC5120618

[ref21] Miller KL , Alfaroalmagro F, Bangerter NK, Thomas DL, Yacoub E, Xu J, Bartsch AJ, Jbabdi S, Sotiropoulos SN, Andersson JLR, et al. 2016. Multimodal population brain imaging in the UK Biobank prospective epidemiological study. Nat Neurosci. 19(11):1523–1536. 27643430 10.1038/nn.4393 PMC5086094

[ref22] Norlander T , Erixon A, Archer T. 2000. Psychological androgyny and creativity: dynamics of gender-role and personality trait. Soc Behav Pers. 28(5):423–436.

[ref23] Nota NM , Burke SM, Den HM, Soleman RS, Lambalk CB, Veltman DJ, Cohenkettenis PT, Kreukels BPC. 2016. Resting state functional connectivity is affected by testosterone treatment in female-to-male transgender persons. Endocrine Abstracts. 41:ep958.

[ref24] Nottelmann ED , Susman EJ, Dorn LD, Inoffgermain G, Loriaux DL, Cutler GB, Chrousos GP. 1987. Developmental processes in early adolescence: relations among chronologic age, pubertal stage, height, weight, and serum levels of gonadotropins, sex steroids, and adrenal androgens. J Adolesc Health Care. 8(3):246–260. 3583875 10.1016/0197-0070(87)90428-1

[ref25] Pauletti RE , Menon M, Cooper PJ, Aults CD, Perry DG. 2017. Psychological androgyny and Children's mental health: a new look with new measures. Sex Roles. 76(11):705–718.

[ref26] Platt JC . 1999. Probabilistic outputs for support vector machines and comparisons to regularized likelihood methods. Adv Large Margin Class. 10(3):61–74.

[ref27] Power JD , Cohen AL, Nelson SM, Wig GS, Barnes KA, Church JA, Vogel AC, Laumann TO, Miezin FM, Schlaggar BL, et al. 2011. Functional network organization of the human brain. Neuron. 72(4):665–678. 22099467 10.1016/j.neuron.2011.09.006 PMC3222858

[ref28] Prakash J , Kotwal ASM, Ryali VSSR, Srivastava K, Bhat P, Shashikumar R. 2010. Does androgyny have psychoprotective attributes? A cross-sectional community-based study. Ind Psychiatry J. 19(2):119–124. 22174535 10.4103/0972-6748.90343 PMC3237128

[ref38b] Rice A . 2006. Gender traits and normative/humanistic behavior. Sociological Viewpoints. 22(2):25–39.

[ref29] Ritchie SJ , Cox SR, Shen X, Lombardo MV, Reus LM, Alloza C, Harris MA, Alderson HL, Hunter S, Neilson E, et al. 2018. Sex differences in the adult human brain: evidence from 5216 UK biobank participants. Cereb Cortex. 28(8):2959–2975. 29771288 10.1093/cercor/bhy109 PMC6041980

[ref30] Rolls ET , Joliot M, Tzouriomazoyer N. 2015. Implementation of a new parcellation of the orbitofrontal cortex in the automated anatomical labeling atlas. NeuroImage. 122:1–5. 26241684 10.1016/j.neuroimage.2015.07.075

[ref31] RongEn F , PaiHsuen C, Chih-Jen L. 2005. Working set selection using second order information for training support vector machines. J Mach Learn Res. 6:1889–1918.

[ref32] Rosario ER , Chang L, Stanczyk FZ, Pike CJ. 2004. Age-related testosterone depletion and the development of Alzheimer disease. JAMA. 292(12):1431–1432. 15383512 10.1001/jama.292.12.1431-b

[ref33] Ruigrok ANV , Salimikhorshidi G, Lai M, Baroncohen S, Lombardo MV, Tait R, Suckling J. 2014. A meta-analysis of sex differences in human brain structure. Neurosci Biobehav Rev. 39(100):34–50. 24374381 10.1016/j.neubiorev.2013.12.004 PMC3969295

[ref34] Satterthwaite TD , Elliott MA, Gerraty RT, Ruparel K, Loughead J, Calkins ME, Eickhoff SB, Hakonarson H, Gur RC, Gur RE, et al. 2013. An improved framework for confound regression and filtering for control of motion artifact in the preprocessing of resting-state functional connectivity data. NeuroImage. 1, 64:240–256. 10.1016/j.neuroimage.2012.08.052 PMC3811142 22926292

[ref35] Satterthwaite TD , Wolf DH, Roalf DR, Ruparel K, Erus G, Vandekar SN, Gennatas ED, Elliott MA, Smith AR, Hakonarson H, et al. 2014. Linked sex differences in cognition and functional connectivity in youth. Cereb Cortex. 25(9):2383–2394. 24646613 10.1093/cercor/bhu036 PMC4537416

[ref36] Schumann G , Loth E, Banaschewski T, Barbot A, Barker GJ, Buchel C, Conrod PJ, Dalley JW, Flor H, Gallinat J, et al. 2010. The IMAGEN study: reinforcement-related behaviour in normal brain function and psychopathology. Mol Psychiatry. 15(12):1128–1139. 21102431 10.1038/mp.2010.4

[ref37a] Smith SM , Beckmann CF, Andersson J, Auerbach EJ, Bijsterbosch J, Douaud G, Duff E, Feinberg DA, Griffanti L, Harms MP, et al. 2013. Resting-state fMRI in the human connectome project. NeuroImage. 80(15):144–168. 23702415 10.1016/j.neuroimage.2013.05.039 PMC3720828

[ref38a] Vafaei A , Alvarado B, Tomás C, Muro C, Martinez B, Zunzunegui MV. 2014. The validity of the 12-item Bem Sex Role Inventory in older Spanish population: An examination of the androgyny model. Arch Gerontol Geriatr. 59(2):257–263. 24997501 10.1016/j.archger.2014.05.012

[ref38] Wang L , Shen H, Tang F, Zang Y, Hu D. 2012. Combined structural and resting-state functional MRI analysis of sexual dimorphism in the young adult human brain: an MVPA approach. NeuroImage. 61(4):931–940. 22498657 10.1016/j.neuroimage.2012.03.080

[ref39] Weis S , Patil K, Hoffstaedter F, Nostro AD, Yeo BTT, Eickhoff SB. 2019. Sex classification by resting state brain connectivity. Cereb Cortex. 30:824–835. 10.1093/cercor/bhz129 PMC7444737 31251328

[ref40] Winkler AM , Ridgway GR, Webster MA, Smith SM, Nichols TE. 2014. Permutation inference for the general linear model. NeuroImage. 92(100):381–397. 24530839 10.1016/j.neuroimage.2014.01.060 PMC4010955

[ref41] Winkler AM , Webster MA, Vidaurre D, Nichols TE, Smith SM. 2015. Multi-level block permutation. NeuroImage. 123:253–268. 26074200 10.1016/j.neuroimage.2015.05.092 PMC4644991

[ref42] Wong YJ , Ho M-HR, Wang S-Y, Miller ISK. 2017. Meta-analyses of the relationship between conformity to masculine norms and mental health-related outcomes. Am Psychol Assoc. doi: 10.1037/cou0000176 27869454

[ref43] Xia M , Wang J, He Y. 2013. BrainNet viewer: a network visualization tool for human brain Connectomics. PLoS One. 8(7):e68910. 10.1371/journal.pone.0068910 PMC3701683 23861951

[ref44] Yao Y , Lu W, Xu B, Li C, Lin C, Waxman D, Feng J. 2013. The increase of the functional entropy of the human brain with age. Sci Rep. 3(1):2853–2853. 24103922 10.1038/srep02853 PMC3793229

