# Supplementary material for: The Human Brain Is Best Described as Being on a Female/Male Continuum: Evidence from a Neuroimaging Connectivity Study
Source: Cereb Cortex. 2021 Jan 20;31(6):3021–33. doi: 10.1093/cercor/bhaa408 (PMC8107794; doi:10.1093/cercor/bhaa408)
Supplement: gender_continuum_supplement_bhaa408 [file gender_continuum_supplement_bhaa408.docx]

**Supplementary Materials**

**The Human Brain is Best Described as Being on a Female/Male Continuum: Evidence from a Neuroimaging Connectivity Study**

***Zhang, Luo, Huang*, *et al.***

Table of Contents

[Methods S1: Quality Control Process 3](#_Toc56527493)

[Methods S2: Validity of gender continuum 4](#_Toc56527494)

[Methods S3: Influence of covariates to SVM classifier 5](#_Toc56527495)

[Methods S4: Sensitivity analysis 6](#_Toc56527496)

[Methods S5: Effect of excluding brain network on classification model for young subjects. 7](#_Toc56527497)

[Methods S6 Influence of additional covariates to sex differences 8](#_Toc56527498)

[Methods S7 Relationship between global signal and global functional connectivity 9](#_Toc56527499)

[Methods S8 Effect of subsampling of training subject from UK Biobank 10](#_Toc56527500)

[Methods S9 The gender continuum and biological sex 11](#_Toc56527501)

[Methods S10 Some details of the pre-processing procedure 12](#_Toc56527502)

[Figure. S1. Network-level sex difference in different parcellations. 13](#_Toc56527503)

[Figure. S2. Stability of gender continuum over 4 HCP runs. 14](#_Toc56527504)

[Figure. S3. Influence of confounding factors to SVM model. 15](#_Toc56527505)

[Figure. S4. sensitivity analysis using cross validation. 16](#_Toc56527506)

[Figure. S5. Corresponding sample size of UK Biobank subjects when changing age upper bound. 17](#_Toc56527507)

[Figure. S6. Influence of Brain network to SVM classifier. 18](#_Toc56527508)

[Table S1. Effect size of sex difference in the brain functional connectivity between and within network. 19](#_Toc56527509)

[Table S2. Effect size of sex difference in the brain functional connectivity between and within network in the YMU dataset. 20](#_Toc56527510)

[Table S3. Female-stronger regional FC sex differences. 21](#_Toc56527511)

[Table S4. Male-stronger regional FC sex differences 22](#_Toc56527512)

[Table S5. t-value of sex difference in the brain functional connectivity between and within network. 23](#_Toc56527513)

[Table S6. Effect size of sex difference in the brain functional connectivity between and within network. 24](#_Toc56527514)

***Methods S1: Quality Control Process***

After pre-processing of the neuroimaging data, we manually checked both the structural and functional image to ensure the quality of data used in our study. We excluded those samples whose brain images either had problems in registration during data pre-processing procedure, or had abnormal signal in multiple cerebral regions, or had severe ghosts caused by head movement. Finally, all subjects with mean FD > 0.3 mm were also excluded from our study. In behaviour analysis, we also excluded subjects who had incomplete family information (including father ID, mother ID, and twin status), and had incomplete total score or sub score of internalizing symptoms.

***Methods S2: Validity of gender continuum***

We tested validity of the gender continuum given by our classification model. The validation of our model was examined to ensure people with intermediate gender continuum were those people who had brain functional network between female brain and male brain rather than noise. We defined 3 subgroups by the score of the brain gender continuum: low score group (gender continuum < 0.35), intermediate score group (0.35 ≤ gender continuum ≤ 0.65), and high score group (gender continuum > 0.65). Totally 66 types of pairwise connections among 11 brain networks (55 inter-network connections and 11 intra-network connections) were analysed. By a one-way ANOVA test, we found 54 out of 66 connections had significant group differences (p<0.05 after Bonferroni Correction), and the intermediate score group always had intermediate connectivity strength. These results suggested that the brains scored intermediately on the gender continuum indeed had the intermediate patterns of functional connectivity rather than noise.

***Methods S3: Influence of covariates to SVM classifier***

We tested the influence of mean FD, SNR and TIV to SVM classifier, where the former two were related to image quality and the last one was related to brain size. We found that when we regressed out all three covariates from the functional connectivity used in training and testing SVM classifier, the test accuracy decreased to 63.56% (AUC=0.6881, Figure S3A).

When we regressed out SNR and mean FD from functional connectivity only, the gender continuum produced by the model was highly correlated with original one with r=0.9583 (df=717, Figure S3B).

***Methods S4: Sensitivity analysis***

When an increased sample size was used for training, the test accuracy increased rapidly when the training sample size was under 2,000 (mean test accuracy ranged from 58.71% to 70.66%). However, when the training sample was larger than about 2,000 subjects, the accuracy increment was markedly slower (mean test accuracy ranged from 72.53% to 76.36%). The difference between those two increasing rates were significant, with 95% CI:0.0063% to 0.0075%. The CV accuracy and test accuracy changed in a similar pattern (r(CV accuracy, test accuracy)=0.9965, for the 15 chosen sample sizes (CV accuracy on UK Biobank were shown in Figure S4A)).

Increasing sample size of training set resulted in improved performance of the classifier on the test set, while the age discordance between training and test set would impair it. Thus, there was a trade-off between a large sample size of the training set and the age accordance between the training set and test set. We adjusted the age upper bound of training subjects used when training the classification model to investigate the influence of both age and sample size. The relationship between upper age bound and test set accuracy were shown in Figure 3J, and the corresponding sample size were shown in Figure S5). We found that with an increased training sample size as well as an increased age difference between subjects from the training and test sets, the test accuracy increased when the upper limit was small (mean accuracy increased from 72.95% to 77.05%) and reached its maximum at approximately 65 years of age. That level of accuracy was maintained as the upper limit increased (mean accuracy=76.74% ± 0.45%; Figure 3J). The results of CV accuracy were shown in Figure S4C-D.

***Methods S5: Effect of excluding brain network on classification model for young subjects.***

We mentioned before that excluding DMN when training classification model on UK Biobank dataset and testing on HCP dataset, would cause the significant decline of prediction accuracy. However, similar results did not reproduce when training and validating on IMAGEN dataset, which implied that functional networks between younger females and younger males did not differ as significant as adults or older subjects. These results were shown in Figure S6.

***Methods S6 Influence of additional covariates to sex differences***

Other than mean FD, SNR and TIV, which we used in main text to examine the influence of confounding factors to sex differences, we additionally used years of education, income and employment status as covariates. These covariates were only available for the HCP dataset, reported by the participants themselves. The Cohen’s d for edge-wise sex differences calculated without considering the covariates was correlated with the Cohen’s d for sex differences calculated controlling mean FD, SNR and TIV, with correlation coefficient of 0.89 (df=4,369). While the former was also correlated with the Cohen’s d for sex differences calculated controlling years of education, income, and employment status, together with mean FD, SNR and TIV, with correlation coefficient of 0.89 (df=4,369). And the Cohen’s d for those two covariates-controlled cases were correlated with correlation coefficient of 1.00.

***Methods S7 Relationship between global signal and global functional connectivity***

We used global functional connectivity in our analysis and identified a nonlinear age-related trajectory of global functional connectivity. While global functional connectivity provided a global description of brain, the global signal also reflected global features of the brain. We found that the global signal is more correlated with regional BOLD signal for those subject with higher global functional connectivity (The averaged correlation coefficients between ROI’s BOLD signal and global signal was correlated with global FC with r = 0.98, df=717). But since the global signal is a time series, and a single time point in the series is meaningless, it cannot be as useful as global functional connectivity to study age related changes.

***Methods S8 Effect of subsampling of training subject from UK Biobank***

The SVM model was trained on the subset of UK Biobank (n=7,400) to maintain the balance of male and female subjects. We recalculated the Cohen’s d for sex differences on that subset, and we found that it was correlated with the Cohen’s d calculated from the entire dataset (n=7,972) with correlation coefficient of 0.9991, which showed that the subsampling procedure does not influence the results of sex differences.

Moreover, if we repeatedly randomly select the training subjects from the UK Biobank, the output gender continuum score on HCP samples was highly correlated with the smallest correlation coefficient of 0.9707 among 100 samplings. Therefore, the random selection of subject does not influence our results.

***Methods S9 The gender continuum and biological sex***

The gender continuum represented the likelihood that the given brain was collected from a male brain. The same gender continuum score could be owned by either a male subject or a female subject. For example, Subject ID 585226 was the female subject with the greatest gender continuum score (i.e. 0.8790). Next, we calculated the group mean of the functional connectivity among the typical male brains (i.e. those male subjects with the gender continuum scores greater than 0.6), and the group mean of the functional connectivity among the typical female brains (i.e. those female subjects with the gender continuum scores less than 0.4). The functional connectivity of Subject 585226 was correlated with the typical male group (r=0.8222) which was significantly stronger than the correlation with the typical female group (r=0.8058), as the 95% confidence interval of the difference between these two correlation coefficients was (0.0047, 0.0281) given by bootstrap. The similar results could also be found for other female subjects with high gender continuum scores or the male subjects with low gender continuum scores. Therefore, a high gender continuum score indicated the brain functional connectivity was correlated more to the typical male brains, regardless of the biological sex of the subject.

***Methods S10 Some details of the pre-processing procedure***

The details of our publicly available pre-processing pipeline can be found at <https://github.com/weikanggong/Resting-state-fMRI-preprocessing>.

In our pre-processing pipeline, T1 images were pre-processed first, and then, functional images were registered to a standard template, followed by regressing out WM and CSF signal and temporal filtering. Finally, the functional imaging data were normalized.

After reorientation, cropping and brain extraction, linear and nonlinear registration tools (FLIRT, and FNIRT) were used to calculate the transformation matrix from the structural T1 images to the standard MNI152 template. The WM and CSF maps were constructed using ‘applywarp’ and ‘fslmaths’ for pre-processing in the next step. Then Boundary-based Registration (BBR) using the function ‘epi_reg’ was used to register the functional images to the T1 images. A transformation matrix from the functional image to the standard template, as well as an inverse transformation matrix from the standard template to the functional image, was computed using ‘convertwarp’ and ‘invwarp’. Then the WM and CSF maps, derived previously, were registered to the functional image using the inverse transformation matrix. The averaged WM and CSF signal was calculated using the ‘3dmaskave’ function and the intersected regions between the probability map and the individual map were found using the ‘fslmaths’ function. Then the ‘3dTproject’ function from AFNI was used to regress out the WM and CSF signal, and the temporal filtering was also completed. Finally, the ‘applywarp’ function was implemented to normalize the pre-processed functional images to 3mm standard space.

**
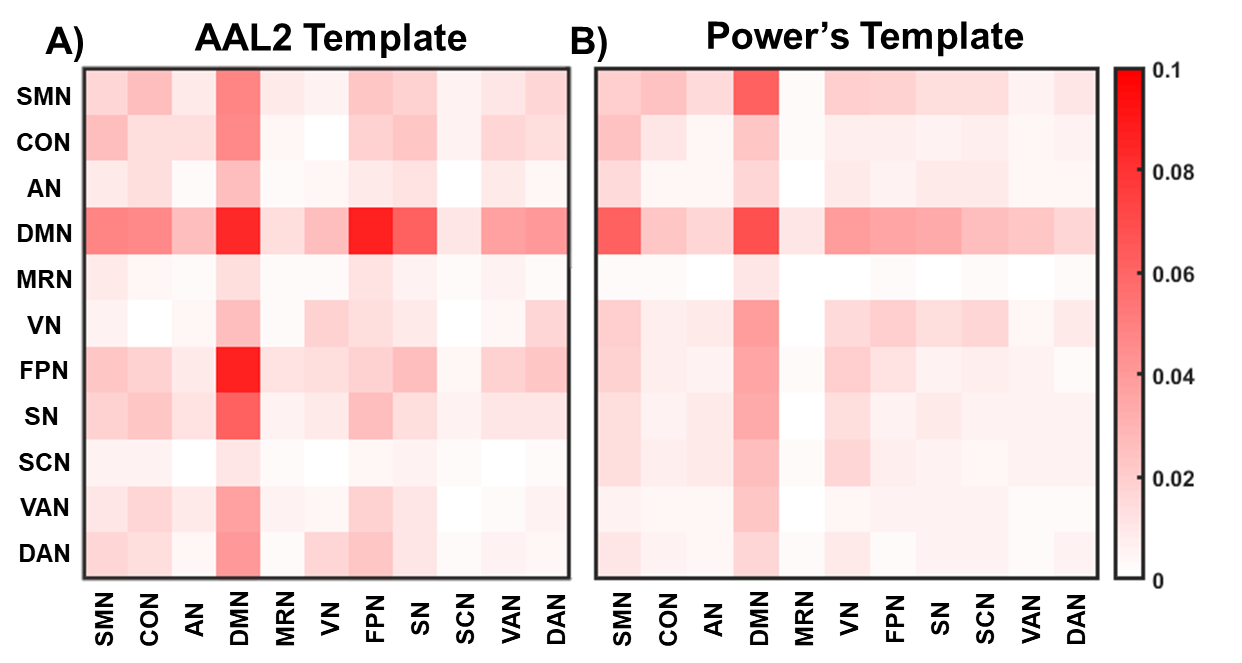
**

**Figure. S1. Network-level sex difference in different parcellations.**

Connections with highest top 5% effect size in **A)** AAL2 parcellation **B)** Power’s parcellation were considered here. The two distribution maps were correlated with r=0.6977; p=3.22×10^-9^ (df=64).


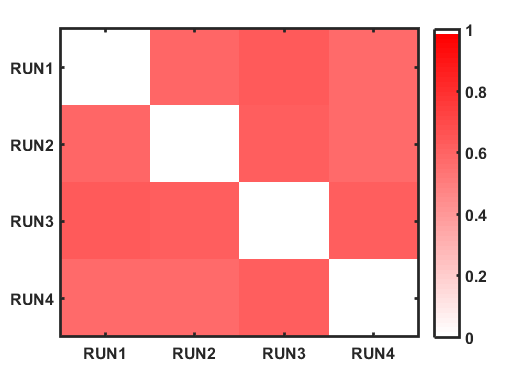


**Figure. S2. Stability of gender continuum over 4 HCP runs.**

The correlation coefficients of gender continuum were shown in the figure. The coefficient ranged from 0.5614 to 0.6242.


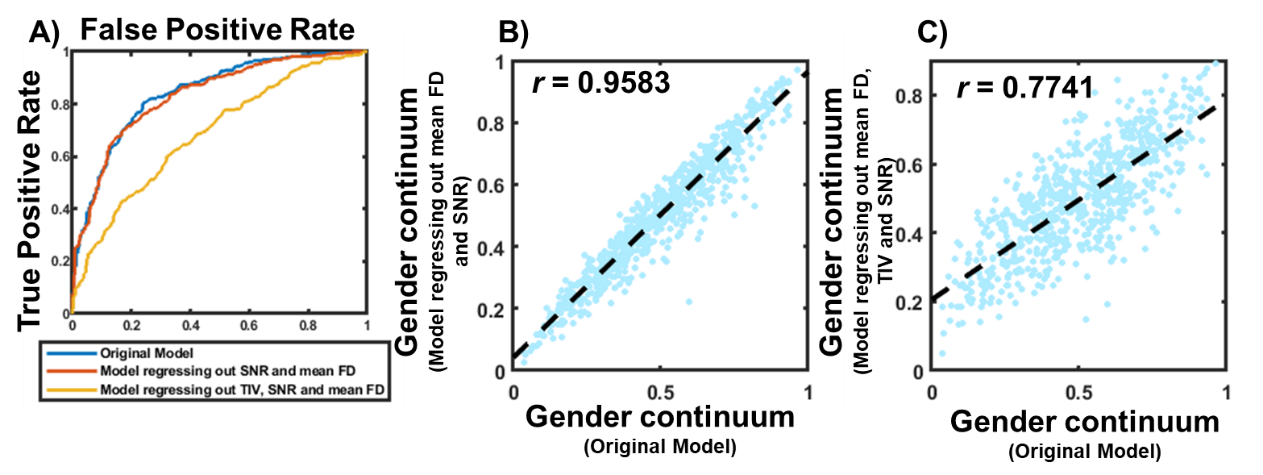


**Figure. S3. Influence of confounding factors to SVM model.**

**A)** ROC of original model (AUC=0.8432), model regressing out SNR and mean FD (AUC=0.8340), and model regressing out TIV, SNR and mean FD (AUC=0.6881) respectively; **B)** Scattered plot of gender continuum calculated from model regressing out B) SNR and mean FD; **C)** TIV, SNR and mean FD versus original model.


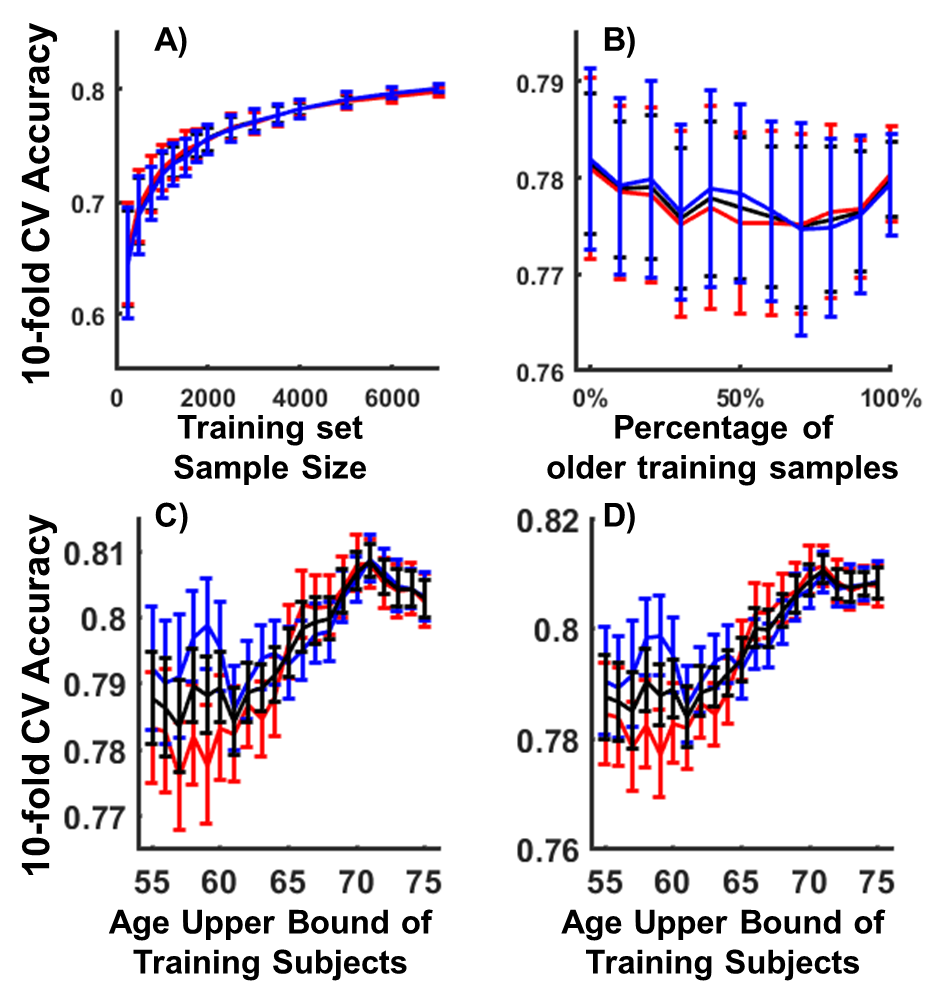


**Figure. S4. sensitivity analysis using cross validation.**

**A)** changing training set sample size; **B)** changing the age composition while fixing the sample size at 3,000; **C)** changing the upper age bound of the participants; **D)** changing the upper age bound when the age-related terms were not regressed out from the model.


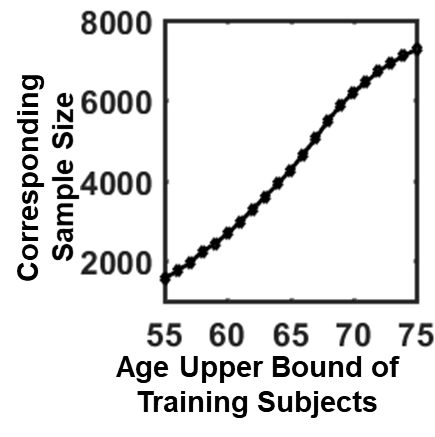


**Figure. S5. Corresponding sample size of UK Biobank subjects when changing age upper bound.**


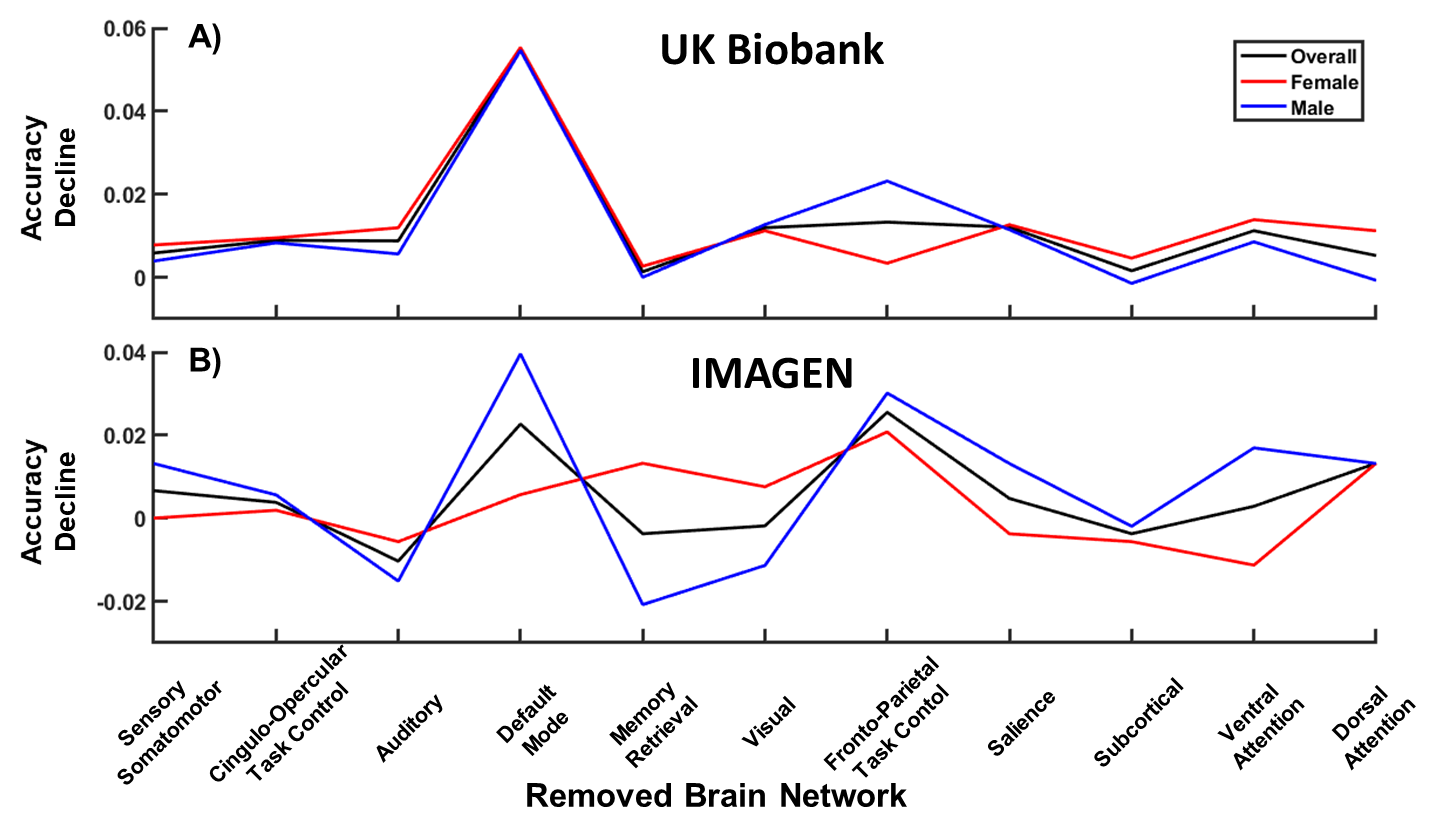


**Figure. S6. Influence of Brain network to SVM classifier.**

Decline of cross-validated classification accuracy of the trained SVM in the **A)** UK biobank; **B)** IMAGEN sample when removing one functional network from input features of the SVM.

**Table S1. Effect size of sex difference in the brain functional connectivity between and within network.**

|  | **Adolescents from IMAGEN** | | | | | | | | | | |
| --- | --- | --- | --- | --- | --- | --- | --- | --- | --- | --- | --- |
|  | **SMN** | **CON** | **AN** | **DMN** | **MRN** | **VN** | **FPN** | **SN** | **SCN** | **VAN** | **DAN** |
| **SMN** | 0.2203 | 0.1937 | 0.1430 | 0.2183 | 0.2414 | 0.1503 | 0.1958 | 0.2415 | 0.1393 | 0.1467 | 0.1989 |
| **CON** | 0.1937 | 0.1853 | 0.1580 | 0.2278 | 0.2323 | 0.1523 | 0.2480 | 0.2622 | 0.1380 | 0.1929 | 0.2502 |
| **AN** | 0.1430 | 0.1580 | 0.0472 | 0.1696 | 0.2680 | 0.0186 | 0.1647 | 0.2373 | 0.1734 | 0.1166 | 0.1361 |
| **DMN** | 0.2183 | 0.2278 | 0.1696 | 0.2076 | 0.1223 | 0.1979 | 0.2355 | 0.2719 | 0.2225 | 0.2256 | 0.2358 |
| **MRN** | 0.2414 | 0.2323 | 0.2680 | 0.1223 | -0.0135 | 0.3371 | 0.1828 | 0.2273 | 0.1239 | 0.2535 | 0.3156 |
| **VN** | 0.1503 | 0.1523 | 0.0186 | 0.1979 | 0.3371 | 0.1060 | 0.1942 | 0.2473 | 0.1560 | 0.0821 | 0.1672 |
| **FPN** | 0.1958 | 0.2480 | 0.1647 | 0.2355 | 0.1828 | 0.1942 | 0.2431 | 0.3057 | 0.2172 | 0.1959 | 0.1785 |
| **SN** | 0.2415 | 0.2622 | 0.2373 | 0.2719 | 0.2273 | 0.2473 | 0.3057 | 0.2968 | 0.2022 | 0.3147 | 0.2906 |
| **SCN** | 0.1393 | 0.1380 | 0.1734 | 0.2225 | 0.1239 | 0.1560 | 0.2172 | 0.2022 | 0.1427 | 0.2202 | 0.1857 |
| **VAN** | 0.1467 | 0.1929 | 0.1166 | 0.2256 | 0.2535 | 0.0821 | 0.1959 | 0.3147 | 0.2202 | 0.1319 | 0.1775 |
| **DAN** | 0.1989 | 0.2502 | 0.1361 | 0.2358 | 0.3156 | 0.1672 | 0.1785 | 0.2906 | 0.1857 | 0.1775 | 0.1947 |
|  | **Adults from HCP** | | | | | | | | | | |
|  | **SMN** | **CON** | **AN** | **DMN** | **MRN** | **VN** | **FPN** | **SN** | **SCN** | **VAN** | **DAN** |
| **SMN** | 0.5865 | 0.5593 | 0.6039 | 0.5799 | 0.5103 | 0.4758 | 0.5646 | 0.5506 | 0.1980 | 0.5300 | 0.5553 |
| **CON** | 0.5593 | 0.6033 | 0.6852 | 0.6434 | 0.5714 | 0.4812 | 0.6287 | 0.6216 | 0.2494 | 0.6115 | 0.5913 |
| **AN** | 0.6039 | 0.6852 | 0.5575 | 0.6725 | 0.7283 | 0.4303 | 0.6724 | 0.7306 | 0.3861 | 0.6116 | 0.6062 |
| **DMN** | 0.5799 | 0.6434 | 0.6725 | 0.5840 | 0.2622 | 0.5319 | 0.5638 | 0.5898 | 0.2648 | 0.6326 | 0.5382 |
| **MRN** | 0.5103 | 0.5714 | 0.7283 | 0.2622 | 0.1638 | 0.5344 | 0.4415 | 0.5131 | 0.1283 | 0.5883 | 0.4742 |
| **VN** | 0.4758 | 0.4812 | 0.4303 | 0.5319 | 0.5344 | 0.4271 | 0.5263 | 0.4966 | 0.1583 | 0.4393 | 0.4854 |
| **FPN** | 0.5646 | 0.6287 | 0.6724 | 0.5638 | 0.4415 | 0.5263 | 0.5533 | 0.6433 | 0.2826 | 0.6128 | 0.5009 |
| **SN** | 0.5506 | 0.6216 | 0.7306 | 0.5898 | 0.5131 | 0.4966 | 0.6433 | 0.6554 | 0.2727 | 0.6338 | 0.5350 |
| **SCN** | 0.1980 | 0.2494 | 0.3861 | 0.2648 | 0.1283 | 0.1583 | 0.2826 | 0.2727 | 0.1820 | 0.2925 | 0.1917 |
| **VAN** | 0.5300 | 0.6115 | 0.6116 | 0.6326 | 0.5883 | 0.4393 | 0.6128 | 0.6338 | 0.2925 | 0.5773 | 0.5777 |
| **DAN** | 0.5553 | 0.5913 | 0.6062 | 0.5382 | 0.4742 | 0.4854 | 0.5009 | 0.5350 | 0.1917 | 0.5777 | 0.5155 |
|  | **Elderlies from UKB** | | | | | | | | | | |
|  | **SMN** | **CON** | **AN** | **DMN** | **MRN** | **VN** | **FPN** | **SN** | **SCN** | **VAN** | **DAN** |
| **SMN** | -0.2045 | -0.2324 | -0.3009 | -0.1698 | -0.1892 | -0.2076 | -0.1267 | -0.1841 | -0.4486 | -0.1991 | -0.1515 |
| **CON** | -0.2324 | -0.3434 | -0.2384 | -0.1264 | -0.2424 | -0.2183 | -0.2355 | -0.2956 | -0.4720 | -0.2209 | -0.1934 |
| **AN** | -0.3009 | -0.2384 | -0.3009 | -0.0908 | -0.0762 | -0.2270 | -0.0950 | -0.2215 | -0.4514 | -0.1411 | -0.1331 |
| **DMN** | -0.1698 | -0.1264 | -0.0908 | -0.3826 | -0.4337 | -0.1731 | -0.3111 | -0.1993 | -0.3673 | -0.1505 | -0.1818 |
| **MRN** | -0.1892 | -0.2424 | -0.0762 | -0.4337 | -0.2794 | -0.0117 | -0.3282 | -0.2160 | -0.3739 | -0.1560 | -0.1964 |
| **VN** | -0.2076 | -0.2183 | -0.2270 | -0.1731 | -0.0117 | -0.2833 | -0.1353 | -0.2010 | -0.2744 | -0.2076 | -0.2143 |
| **FPN** | -0.1267 | -0.2355 | -0.0950 | -0.3111 | -0.3282 | -0.1353 | -0.3505 | -0.2374 | -0.3736 | -0.1997 | -0.2407 |
| **SN** | -0.1841 | -0.2956 | -0.2215 | -0.1993 | -0.2160 | -0.2010 | -0.2374 | -0.3018 | -0.4373 | -0.1950 | -0.1998 |
| **SCN** | -0.4486 | -0.4720 | -0.4514 | -0.3673 | -0.3739 | -0.2744 | -0.3736 | -0.4373 | -0.2089 | -0.4668 | -0.4003 |
| **VAN** | -0.1991 | -0.2209 | -0.1411 | -0.1505 | -0.1560 | -0.2076 | -0.1997 | -0.1950 | -0.4668 | -0.1760 | -0.1731 |
| **DAN** | -0.1515 | -0.1934 | -0.1331 | -0.1818 | -0.1964 | -0.2143 | -0.2407 | -0.1998 | -0.4003 | -0.1731 | -0.1907 |

**NOTE**: Cohen’s d was reported for the sex difference. When the network connectivity was stronger in males than that in females, a positive effect size was observed. Abbreviations: SMN--Sensory/Somatomotor Network; CON--Cingulo-Opercular Task Control Network; AN--Auditory Network; DMN--Default Mode Network; MRN--Memory Retrieval Network; VN--Visual Network; FPN--Frontal-Parietal Task Control Network; SN--Salience Network; SCN--Subcortical Network; VAN--Ventral Attention Network; DAN--Dorsal Attention Network.

**Table S2. Effect size of sex difference in the brain functional connectivity between and within network in the YMU dataset.**

| **Age < 45 years old** | | | | | | | | | | | |
| --- | --- | --- | --- | --- | --- | --- | --- | --- | --- | --- | --- |
|  | **SMN** | **CON** | **AN** | **DMN** | **MRN** | **VN** | **FPN** | **SN** | **SCN** | **VAN** | **DAN** |
| **SMN** | 0.2448 | 0.4474 | 0.2693 | 0.5183 | 0.4802 | 0.2429 | 0.5949 | 0.5366 | 0.4567 | 0.4984 | 0.4832 |
| **CON** | 0.4474 | 0.4446 | 0.3175 | 0.5600 | 0.4264 | 0.3912 | 0.5559 | 0.5227 | 0.3487 | 0.4678 | 0.4972 |
| **AN** | 0.2693 | 0.3175 | 0.0920 | 0.4042 | 0.3258 | -0.0323 | 0.4834 | 0.4532 | 0.5666 | 0.4973 | 0.3903 |
| **DMN** | 0.5183 | 0.5600 | 0.4042 | 0.6501 | 0.3427 | 0.4805 | 0.6108 | 0.5747 | 0.4041 | 0.5106 | 0.4184 |
| **MRN** | 0.4802 | 0.4264 | 0.3258 | 0.3427 | 0.1779 | 0.2224 | 0.4135 | 0.4253 | -0.0099 | 0.4034 | 0.1337 |
| **VN** | 0.2429 | 0.3912 | -0.0323 | 0.4805 | 0.2224 | -0.0979 | 0.5658 | 0.4717 | 0.4035 | 0.3781 | 0.3727 |
| **FPN** | 0.5949 | 0.5559 | 0.4834 | 0.6108 | 0.4135 | 0.5658 | 0.5960 | 0.5691 | 0.4331 | 0.5422 | 0.5907 |
| **SN** | 0.5366 | 0.5227 | 0.4532 | 0.5747 | 0.4253 | 0.4717 | 0.5691 | 0.4888 | 0.2693 | 0.5908 | 0.5473 |
| **SCN** | 0.4567 | 0.3487 | 0.5666 | 0.4041 | -0.0099 | 0.4035 | 0.4331 | 0.2693 | 0.2256 | 0.4729 | 0.5028 |
| **VAN** | 0.4984 | 0.4678 | 0.4973 | 0.5106 | 0.4034 | 0.3781 | 0.5422 | 0.5908 | 0.4729 | 0.5080 | 0.5630 |
| **DAN** | 0.4832 | 0.4972 | 0.3903 | 0.4184 | 0.1337 | 0.3727 | 0.5907 | 0.5473 | 0.5028 | 0.5630 | 0.4029 |
| **Age** $\boldsymbol{\geq}$ **45 years old** | | | | | | | | | | | |
|  | **SMN** | **CON** | **AN** | **DMN** | **MRN** | **VN** | **FPN** | **SN** | **SCN** | **VAN** | **DAN** |
| **SMN** | 0.1340 | -0.2009 | -0.0649 | -0.2013 | 0.1544 | 0.0438 | -0.3165 | -0.5815 | -0.4932 | -0.1903 | 0.0141 |
| **CON** | -0.2009 | -0.2811 | -0.2905 | -0.1212 | 0.1898 | -0.4181 | -0.3036 | -0.5170 | -0.3840 | -0.4126 | -0.2601 |
| **AN** | -0.0649 | -0.2905 | -0.2052 | -0.0623 | 0.3553 | -0.3237 | -0.0490 | -0.3328 | -0.3454 | -0.1883 | 0.0883 |
| **DMN** | -0.2013 | -0.1212 | -0.0623 | -0.3932 | 0.3204 | -0.2111 | -0.5510 | -0.4954 | -0.5241 | -0.4144 | -0.3129 |
| **MRN** | 0.1544 | 0.1898 | 0.3553 | 0.3204 | -0.0038 | 0.5010 | -0.2300 | -0.1027 | -0.1215 | -0.0208 | 0.0410 |
| **VN** | 0.0438 | -0.4181 | -0.3237 | -0.2111 | 0.5010 | 0.3534 | -0.3966 | -0.6602 | -0.6943 | -0.4254 | 0.1814 |
| **FPN** | -0.3165 | -0.3036 | -0.0490 | -0.5510 | -0.2300 | -0.3966 | -0.5576 | -0.4916 | -0.5408 | -0.2771 | -0.6703 |
| **SN** | -0.5815 | -0.5170 | -0.3328 | -0.4954 | -0.1027 | -0.6602 | -0.4916 | -0.5210 | -0.4298 | -0.5738 | -0.7138 |
| **SCN** | -0.4932 | -0.3840 | -0.3454 | -0.5241 | -0.1215 | -0.6943 | -0.5408 | -0.4298 | -0.4030 | -0.5847 | -0.5982 |
| **VAN** | -0.1903 | -0.4126 | -0.1883 | -0.4144 | -0.0208 | -0.4254 | -0.2771 | -0.5738 | -0.5847 | -0.3524 | -0.2701 |
| **DAN** | 0.0141 | -0.2601 | 0.0883 | -0.3129 | 0.0410 | 0.1814 | -0.6703 | -0.7138 | -0.5982 | -0.2701 | -0.0135 |

**Table S3. Female-stronger regional FC sex differences.**

| **IMAGEN** | | **HCP** | | **UK Biobank** | |
| --- | --- | --- | --- | --- | --- |
| **ROI** | **d** | **ROI** | **d** | **ROI** | **d** |
| Cingulate_Post_R | -0.0537 | Cingulate_Post_R | -0.0724 | Thalamus_L | -0.1287 |
| Precuneus_L | -0.0481 | Angular_R | -0.0635 | Thalamus_R | -0.1234 |
| Cingulate_Post_L | -0.0441 | Frontal_Sup_Med_L | -0.0581 | Frontal_Sup_Med_L | -0.1158 |
| Precuneus_R | -0.0401 | Precuneus_L | -0.0569 | Frontal_Sup_2_L | -0.1099 |
| Frontal_Sup_Med_R | -0.0354 | Cingulate_Post_L | -0.0567 | Angular_R | -0.1095 |
| Rectus_L | -0.0343 | Frontal_Med_Orb_R | -0.0527 | Frontal_Sup_Med_R | -0.1071 |
| Frontal_Med_Orb_L | -0.0328 | Rectus_L | -0.0521 | Frontal_Sup_2_R | -0.1065 |
| Olfactory_L | -0.0316 | Precuneus_R | -0.0491 | ParaHippocampal_L | -0.1050 |
| Angular_R | -0.0313 | Frontal_Med_Orb_L | -0.0487 | Precentral_R | -0.1047 |

**NOTE**: 10 ROIs (regions of interest) with largest averaged effect size of sex differences where female had stronger connections were listed in the table. Abbreviations: L—left, R—right, Sup—superior, Post—Posterior, Med—Medial, and Orb—orbital.

**Table S4. Male-stronger regional FC sex differences**

| **IMAGEN** | | **HCP** | | **UK Biobank** | |
| --- | --- | --- | --- | --- | --- |
| **ROI** | **d** | **ROI** | **d** | **ROI** | **d** |
| Cingulate_Ant_L | 0.0977 | Insula_L | 0.2469 | Angular_L | 0.0931 |
| Cingulate_Ant_R | 0.0910 | Heschl_R | 0.2468 | Angular_R | 0.0926 |
| Insula_L | 0.0883 | Temporal_Pole_Sup_R | 0.2452 | Precuneus_L | 0.0699 |
| Cingulate_Mid_L | 0.0878 | Rolandic_Oper_L | 0.2432 | Precuneus_R | 0.0667 |
| Frontal_Mid_2_R | 0.0873 | Insula_R | 0.2414 | Parietal_Inf_R | 0.0647 |
| Parietal_Sup_R | 0.0857 | Rolandic_Oper_R | 0.2391 | Occipital_Mid_R | 0.0594 |
| Insula_R | 0.0854 | Temporal_Sup_L | 0.2384 | Frontal_Inf_Orb_2_R | 0.0565 |
| Precuneus_L | 0.0845 | Cingulate_Mid_L | 0.2333 | Parietal_Inf_L | 0.0560 |
| Frontal_Mid_2_L | 0.0844 | Heschl_L | 0.2325 | Lingual_R | 0.0553 |

**NOTE**: 10 ROIs (regions of interest) with largest averaged effect size of sex differences where female had stronger connections were listed in the table. Abbreviations: Ant—anterior, Mid—middle, Sup – superior, Oper—operculum, Orb – orbital.

| **Adolescents from IMAGEN (df=711)** | | | | | | | | | | | |
| --- | --- | --- | --- | --- | --- | --- | --- | --- | --- | --- | --- |
|  | **SMN** | **CON** | **AN** | **DMN** | **MRN** | **VN** | **FPN** | **SN** | **SCN** | **VAN** | **DAN** |
| **SMN** | 3.0957 | 2.7217 | 2.0096 | 3.0673 | 3.3927 | 2.1117 | 2.7515 | 3.3933 | 1.9572 | 2.0620 | 2.7955 |
| **CON** | 2.7217 | 2.6040 | 2.2200 | 3.2003 | 3.2645 | 2.1395 | 3.4841 | 3.6848 | 1.9390 | 2.7099 | 3.5152 |
| **AN** | 2.0096 | 2.2200 | 0.6626 | 2.3827 | 3.7665 | 0.2618 | 2.3146 | 3.3341 | 2.4361 | 1.6390 | 1.9120 |
| **DMN** | 3.0673 | 3.2003 | 2.3827 | 2.9166 | 1.7184 | 2.7801 | 3.3085 | 3.8200 | 3.1271 | 3.1699 | 3.3131 |
| **MRN** | 3.3927 | 3.2645 | 3.7665 | 1.7184 | -0.1898 | 4.7371 | 2.5681 | 3.1933 | 1.7407 | 3.5624 | 4.4351 |
| **VN** | 2.1117 | 2.1395 | 0.2618 | 2.7801 | 4.7371 | 1.4901 | 2.7295 | 3.4752 | 2.1918 | 1.1537 | 2.3488 |
| **FPN** | 2.7515 | 3.4841 | 2.3146 | 3.3085 | 2.5681 | 2.7295 | 3.4155 | 4.2959 | 3.0521 | 2.7533 | 2.5086 |
| **SN** | 3.3933 | 3.6848 | 3.3341 | 3.8200 | 3.1933 | 3.4752 | 4.2959 | 4.1706 | 2.8414 | 4.4223 | 4.0834 |
| **SCN** | 1.9572 | 1.9390 | 2.4361 | 3.1271 | 1.7407 | 2.1918 | 3.0521 | 2.8414 | 2.0046 | 3.0948 | 2.6094 |
| **VAN** | 2.0620 | 2.7099 | 1.6390 | 3.1699 | 3.5624 | 1.1537 | 2.7533 | 4.4223 | 3.0948 | 1.8539 | 2.4944 |
| **DAN** | 2.7955 | 3.5152 | 1.9120 | 3.3131 | 4.4351 | 2.3488 | 2.5086 | 4.0834 | 2.6094 | 2.4944 | 2.7364 |
| **Adults from HCP (df=717)** | | | | | | | | | | | |
|  | **SMN** | **CON** | **AN** | **DMN** | **MRN** | **VN** | **FPN** | **SN** | **SCN** | **VAN** | **DAN** |
| **SMN** | 7.8140 | 7.4512 | 8.0458 | 7.7266 | 6.7990 | 6.3396 | 7.5217 | 7.3355 | 2.6376 | 7.0609 | 7.3976 |
| **CON** | 7.4512 | 8.0374 | 9.1285 | 8.5722 | 7.6129 | 6.4117 | 8.3757 | 8.2816 | 3.3232 | 8.1473 | 7.8775 |
| **AN** | 8.0458 | 9.1285 | 7.4282 | 8.9600 | 9.7030 | 5.7326 | 8.9585 | 9.7343 | 5.1437 | 8.1482 | 8.0760 |
| **DMN** | 7.7266 | 8.5722 | 8.9600 | 7.7811 | 3.4927 | 7.0871 | 7.5114 | 7.8573 | 3.5273 | 8.4277 | 7.1709 |
| **MRN** | 6.7990 | 7.6129 | 9.7030 | 3.4927 | 2.1817 | 7.1193 | 5.8823 | 6.8364 | 1.7095 | 7.8374 | 6.3173 |
| **VN** | 6.3396 | 6.4117 | 5.7326 | 7.0871 | 7.1193 | 5.6904 | 7.0124 | 6.6163 | 2.1095 | 5.8523 | 6.4666 |
| **FPN** | 7.5217 | 8.3757 | 8.9585 | 7.5114 | 5.8823 | 7.0124 | 7.3711 | 8.5707 | 3.7657 | 8.1638 | 6.6741 |
| **SN** | 7.3355 | 8.2816 | 9.7343 | 7.8573 | 6.8364 | 6.6163 | 8.5707 | 8.7323 | 3.6331 | 8.4446 | 7.1276 |
| **SCN** | 2.6376 | 3.3232 | 5.1437 | 3.5273 | 1.7095 | 2.1095 | 3.7657 | 3.6331 | 2.4247 | 3.8974 | 2.5537 |
| **VAN** | 7.0609 | 8.1473 | 8.1482 | 8.4277 | 7.8374 | 5.8523 | 8.1638 | 8.4446 | 3.8974 | 7.6916 | 7.6961 |
| **DAN** | 7.3976 | 7.8775 | 8.0760 | 7.1709 | 6.3173 | 6.4666 | 6.6741 | 7.1276 | 2.5537 | 7.6961 | 6.8678 |
| **Elderlies from UKB (df=7970)** | | | | | | | | | | | |
|  | **SMN** | **CON** | **AN** | **DMN** | **MRN** | **VN** | **FPN** | **SN** | **SCN** | **VAN** | **DAN** |
| **SMN** | -9.1083 | -10.3518 | -13.4064 | -7.5646 | -8.4269 | -9.2465 | -5.6421 | -8.2005 | -19.9818 | -8.8708 | -6.7486 |
| **CON** | -10.3518 | -15.2985 | -10.6187 | -5.6301 | -10.7974 | -9.7239 | -10.4894 | -13.1672 | -21.0254 | -9.8410 | -8.6159 |
| **AN** | -13.4064 | -10.6187 | -13.4025 | -4.0430 | -3.3964 | -10.1139 | -4.2315 | -9.8681 | -20.1095 | -6.2849 | -5.9293 |
| **DMN** | -7.5646 | -5.6301 | -4.0430 | -17.0433 | -19.3200 | -7.7105 | -13.8588 | -8.8801 | -16.3613 | -6.7028 | -8.1006 |
| **MRN** | -8.4269 | -10.7974 | -3.3964 | -19.3200 | -12.4477 | -0.5212 | -14.6203 | -9.6206 | -16.6559 | -6.9489 | -8.7501 |
| **VN** | -9.2465 | -9.7239 | -10.1139 | -7.7105 | -0.5212 | -12.6218 | -6.0263 | -8.9550 | -12.2221 | -9.2478 | -9.5481 |
| **FPN** | -5.6421 | -10.4894 | -4.2315 | -13.8588 | -14.6203 | -6.0263 | -15.6149 | -10.5766 | -16.6433 | -8.8942 | -10.7211 |
| **SN** | -8.2005 | -13.1672 | -9.8681 | -8.8801 | -9.6206 | -8.9550 | -10.5766 | -13.4427 | -19.4823 | -8.6861 | -8.9022 |
| **SCN** | -19.9818 | -21.0254 | -20.1095 | -16.3613 | -16.6559 | -12.2221 | -16.6433 | -19.4823 | -9.3074 | -20.7946 | -17.8329 |
| **VAN** | -8.8708 | -9.8410 | -6.2849 | -6.7028 | -6.9489 | -9.2478 | -8.8942 | -8.6861 | -20.7946 | -7.8401 | -7.7132 |
| **DAN** | -6.7486 | -8.6159 | -5.9293 | -8.1006 | -8.7501 | -9.5481 | -10.7211 | -8.9022 | -17.8329 | -7.7132 | -8.4932 |

**Table S5. t-value of sex difference in the brain functional connectivity between and within network.**

|  | **Adolescents from IMAGEN (df=711)** | | | | | | | | | | |
| --- | --- | --- | --- | --- | --- | --- | --- | --- | --- | --- | --- |
|  | **SMN** | **CON** | **AN** | **DMN** | **MRN** | **VN** | **FPN** | **SN** | **SCN** | **VAN** | **DAN** |
| **SMN** | 2.03E-03 | 6.64E-03 | 4.48E-02 | 2.23E-03 | 7.27E-04 | 3.50E-02 | 6.07E-03 | 7.25E-04 | 5.07E-02 | 3.95E-02 | 5.31E-03 |
| **CON** | 6.64E-03 | 9.39E-03 | 2.67E-02 | 1.43E-03 | 1.14E-03 | 3.27E-02 | 5.21E-04 | 2.45E-04 | 5.29E-02 | 6.88E-03 | 4.65E-04 |
| **AN** | 4.48E-02 | 2.67E-02 | 5.08E-01 | 1.74E-02 | 1.78E-04 | 7.94E-01 | 2.09E-02 | 8.96E-04 | 1.51E-02 | 1.02E-01 | 5.62E-02 |
| **DMN** | 2.23E-03 | 1.43E-03 | 1.74E-02 | 3.64E-03 | 8.61E-02 | 5.56E-03 | 9.81E-04 | 1.44E-04 | 1.83E-03 | 1.58E-03 | 9.65E-04 |
| **MRN** | 7.27E-04 | 1.14E-03 | 1.78E-04 | 8.61E-02 | 8.50E-01 | 2.57E-06 | 1.04E-02 | 1.46E-03 | 8.21E-02 | 3.90E-04 | 1.05E-05 |
| **VN** | 3.50E-02 | 3.27E-02 | 7.94E-01 | 5.56E-03 | 2.57E-06 | 1.37E-01 | 6.48E-03 | 5.39E-04 | 2.87E-02 | 2.49E-01 | 1.91E-02 |
| **FPN** | 6.07E-03 | 5.21E-04 | 2.09E-02 | 9.81E-04 | 1.04E-02 | 6.48E-03 | 6.69E-04 | 1.96E-05 | 2.35E-03 | 6.04E-03 | 1.23E-02 |
| **SN** | 7.25E-04 | 2.45E-04 | 8.96E-04 | 1.44E-04 | 1.46E-03 | 5.39E-04 | 1.96E-05 | 3.38E-05 | 4.61E-03 | 1.11E-05 | 4.89E-05 |
| **SCN** | 5.07E-02 | 5.29E-02 | 1.51E-02 | 1.83E-03 | 8.21E-02 | 2.87E-02 | 2.35E-03 | 4.61E-03 | 4.53E-02 | 2.04E-03 | 9.24E-03 |
| **VAN** | 3.95E-02 | 6.88E-03 | 1.02E-01 | 1.58E-03 | 3.90E-04 | 2.49E-01 | 6.04E-03 | 1.11E-05 | 2.04E-03 | 6.41E-02 | 1.28E-02 |
| **DAN** | 5.31E-03 | 4.65E-04 | 5.62E-02 | 9.65E-04 | 1.05E-05 | 1.91E-02 | 1.23E-02 | 4.89E-05 | 9.24E-03 | 1.28E-02 | 6.35E-03 |
|  | **Adults from HCP (df=717)** | | | | | | | | | | |
|  | **SMN** | **CON** | **AN** | **DMN** | **MRN** | **VN** | **FPN** | **SN** | **SCN** | **VAN** | **DAN** |
| **SMN** | 1.97E-14 | 2.67E-13 | 3.55E-15 | 3.73E-14 | 2.22E-11 | 4.07E-10 | 1.62E-13 | 5.99E-13 | 8.53E-03 | 3.92E-12 | 3.88E-13 |
| **CON** | 2.67E-13 | 3.78E-15 | 6.95E-19 | 6.22E-17 | 8.46E-14 | 2.61E-10 | 2.88E-16 | 5.95E-16 | 9.35E-04 | 1.65E-15 | 1.24E-14 |
| **AN** | 3.55E-15 | 6.95E-19 | 3.14E-13 | 2.77E-18 | 5.35E-21 | 1.46E-08 | 2.81E-18 | 4.08E-21 | 3.48E-07 | 1.64E-15 | 2.83E-15 |
| **DMN** | 3.73E-14 | 6.22E-17 | 2.77E-18 | 2.51E-14 | 5.07E-04 | 3.28E-12 | 1.74E-13 | 1.44E-14 | 4.47E-04 | 1.93E-16 | 1.86E-12 |
| **MRN** | 2.22E-11 | 8.46E-14 | 5.35E-21 | 5.07E-04 | 2.95E-02 | 2.64E-12 | 6.21E-09 | 1.74E-11 | 8.78E-02 | 1.66E-14 | 4.67E-10 |
| **VN** | 4.07E-10 | 2.61E-10 | 1.46E-08 | 3.28E-12 | 2.64E-12 | 1.85E-08 | 5.42E-12 | 7.21E-11 | 3.52E-02 | 7.37E-09 | 1.85E-10 |
| **FPN** | 1.62E-13 | 2.88E-16 | 2.81E-18 | 1.74E-13 | 6.21E-09 | 5.42E-12 | 4.68E-13 | 6.29E-17 | 1.80E-04 | 1.46E-15 | 4.98E-11 |
| **SN** | 5.99E-13 | 5.95E-16 | 4.08E-21 | 1.44E-14 | 1.74E-11 | 7.21E-11 | 6.29E-17 | 1.74E-17 | 3.00E-04 | 1.69E-16 | 2.50E-12 |
| **SCN** | 8.53E-03 | 9.35E-04 | 3.48E-07 | 4.47E-04 | 8.78E-02 | 3.52E-02 | 1.80E-04 | 3.00E-04 | 1.56E-02 | 1.06E-04 | 1.09E-02 |
| **VAN** | 3.92E-12 | 1.65E-15 | 1.64E-15 | 1.93E-16 | 1.66E-14 | 7.37E-09 | 1.46E-15 | 1.69E-16 | 1.06E-04 | 4.81E-14 | 4.65E-14 |
| **DAN** | 3.88E-13 | 1.24E-14 | 2.83E-15 | 1.86E-12 | 4.67E-10 | 1.85E-10 | 4.98E-11 | 2.50E-12 | 1.09E-02 | 4.65E-14 | 1.41E-11 |
|  | **Elderlies from UKB (df=7970)** | | | | | | | | | | |
|  | **SMN** | **CON** | **AN** | **DMN** | **MRN** | **VN** | **FPN** | **SN** | **SCN** | **VAN** | **DAN** |
| **SMN** | 1.04E-19 | 5.91E-25 | 1.52E-40 | 4.32E-14 | 4.17E-17 | 2.93E-20 | 1.74E-08 | 2.77E-16 | 1.03E-86 | 8.85E-19 | 1.60E-11 |
| **CON** | 5.91E-25 | 4.28E-52 | 3.65E-26 | 1.86E-08 | 5.44E-27 | 3.17E-22 | 1.42E-25 | 3.47E-39 | 1.46E-95 | 1.01E-22 | 8.28E-18 |
| **AN** | 1.52E-40 | 3.65E-26 | 1.60E-40 | 5.33E-05 | 6.86E-04 | 6.68E-24 | 2.35E-05 | 7.74E-23 | 8.93E-88 | 3.45E-10 | 3.17E-09 |
| **DMN** | 4.32E-14 | 1.86E-08 | 5.33E-05 | 5.28E-64 | 2.59E-81 | 1.40E-14 | 3.56E-43 | 8.15E-19 | 3.31E-59 | 2.18E-11 | 6.28E-16 |
| **MRN** | 4.17E-17 | 5.44E-27 | 6.86E-04 | 2.59E-81 | 3.05E-35 | 6.02E-01 | 8.64E-48 | 8.59E-22 | 2.95E-61 | 3.97E-12 | 2.57E-18 |
| **VN** | 2.93E-20 | 3.17E-22 | 6.68E-24 | 1.40E-14 | 6.02E-01 | 3.55E-36 | 1.75E-09 | 4.17E-19 | 4.77E-34 | 2.89E-20 | 1.72E-21 |
| **FPN** | 1.74E-08 | 1.42E-25 | 2.35E-05 | 3.56E-43 | 8.64E-48 | 1.75E-09 | 3.64E-54 | 5.68E-26 | 3.62E-61 | 7.19E-19 | 1.23E-26 |
| **SN** | 2.77E-16 | 3.47E-39 | 7.74E-23 | 8.15E-19 | 8.59E-22 | 4.17E-19 | 5.68E-26 | 9.43E-41 | 1.27E-82 | 4.50E-18 | 6.69E-19 |
| **SCN** | 1.03E-86 | 1.46E-95 | 8.93E-88 | 3.31E-59 | 2.95E-61 | 4.77E-34 | 3.62E-61 | 1.27E-82 | 1.66E-20 | 1.43E-93 | 8.80E-70 |
| **VAN** | 8.85E-19 | 1.01E-22 | 3.45E-10 | 2.18E-11 | 3.97E-12 | 2.89E-20 | 7.19E-19 | 4.50E-18 | 1.43E-93 | 5.09E-15 | 1.38E-14 |
| **DAN** | 1.60E-11 | 8.28E-18 | 3.17E-09 | 6.28E-16 | 2.57E-18 | 1.72E-21 | 1.23E-26 | 6.69E-19 | 8.80E-70 | 1.38E-14 | 2.37E-17 |

**Table S6. Effect size of sex difference in the brain functional connectivity between and within network.**
